# Comparison of single-molecule sequencing and hybrid approaches for finishing the genome of *Clostridium autoethanogenum* and analysis of CRISPR systems in industrial relevant Clostridia

**DOI:** 10.1186/1754-6834-7-40

**Published:** 2014-03-21

**Authors:** Steven D Brown, Shilpa Nagaraju, Sagar Utturkar, Sashini De Tissera, Simón Segovia, Wayne Mitchell, Miriam L Land, Asela Dassanayake, Michael Köpke

**Affiliations:** 1 Biosciences Division, Oak Ridge National Laboratory, Oak Ridge, TN 37831, USA; 2 BioEnergy Science Center, Oak Ridge National Laboratory, Oak Ridge, TN 37831, USA; 3 Graduate School of Genome Science and Technology, University of Tennessee, Knoxville, TN 37996, USA; 4 LanzaTech NZ, Ltd., Auckland, New Zealand

## Abstract

**Background:**

*Clostridium autoethanogenum* strain JA1-1 (DSM 10061) is an acetogen capable of fermenting CO, CO_2_ and H_2_ (e.g. from syngas or waste gases) into biofuel ethanol and commodity chemicals such as 2,3-butanediol. A draft genome sequence consisting of 100 contigs has been published.

**Results:**

A closed, high-quality genome sequence for *C. autoethanogenum* DSM10061 was generated using only the latest single-molecule DNA sequencing technology and without the need for manual finishing. It is assigned to the most complex genome classification based upon genome features such as repeats, prophage, nine copies of the rRNA gene operons. It has a low G + C content of 31.1%. Illumina, 454, Illumina/454 hybrid assemblies were generated and then compared to the draft and PacBio assemblies using summary statistics, CGAL, QUAST and REAPR bioinformatics tools and comparative genomic approaches. Assemblies based upon shorter read DNA technologies were confounded by the large number repeats and their size, which in the case of the rRNA gene operons were ~5 kb. CRISPR (Clustered Regularly Interspaced Short Paloindromic Repeats) systems among biotechnologically relevant Clostridia were classified and related to plasmid content and prophages. Potential associations between plasmid content and CRISPR systems may have implications for historical industrial scale Acetone-Butanol-Ethanol (ABE) fermentation failures and future large scale bacterial fermentations. While *C. autoethanogenum* contains an active CRISPR system, no such system is present in the closely related *Clostridium ljungdahlii* DSM 13528. A common prophage inserted into the Arg-tRNA shared between the strains suggests a common ancestor. However, *C. ljungdahlii* contains several additional putative prophages and it has more than double the amount of prophage DNA compared to *C. autoethanogenum*. Other differences include important metabolic genes for central metabolism (as an additional hydrogenase and the absence of a phophoenolpyruvate synthase) and substrate utilization pathway (mannose and aromatics utilization) that might explain phenotypic differences between *C. autoethanogenum* and *C. ljungdahlii*.

**Conclusions:**

Single molecule sequencing will be increasingly used to produce finished microbial genomes. The complete genome will facilitate comparative genomics and functional genomics and support future comparisons between Clostridia and studies that examine the evolution of plasmids, bacteriophage and CRISPR systems.

## Background

The development of next-generation DNA sequencing technologies since the first human genome sequence was completed has led to remarkable increases in sequencing efficiency on the order of approximately 100,000-fold [1]. Costs have dropped dramatically and computational methods have advanced along with sequencing technology, leading to large increases in DNA sequencing output and in the number of available genome sequences [2,3]. A variety of assembly algorithms and methods for quality evaluation have been developed [3-13]. However, the majority of sequenced genomes are incomplete due to technical difficulties, time, and expense leading to an increasing disparity between the number of finished and draft genomes in databases [1-3,5,14].

The PacBio sequencing system [15] is the only long-read, single-molecule sequencer available at present and the performance of the PacBio RS system was compared to two short-read sequencing platforms also released in 2011 [16]. The original RS system with C1 chemistry generated mean read-lengths in the range of 1,500 bp and yielded approximately 100 Mb of sequence data per run, and reads in this range were useful in generating improved scaffolds for *de novo* assemblies. However, the original system was not optimal for *de novo* assembly applications [16] and hybrid assembly approaches have been developed to overcome limitations in short-read technologies and higher error rates associated with third-generation technology [17,18].

Repetitive stretches of DNA are abundant and are one of the main technical challenges that hinder accurate sequencing and genome assembly efforts [1]. In the case of bacteria, the rRNA gene operon is often the largest region of repetitive sequence and range in size between 5 and 7 kb [19]. Last year, the longest PacBio RS reads were reported as being approximately 14 kb and these longer reads are useful in resolving repeats during genome assemblies [3]. The PacBio RS II system was released last year and it produces more and longer reads. In a recent study, the longest read before correction was 15,634 bp and the genomes of six bacteria were sequenced and assembled using single-molecule sequencing based on C2 chemistry [14]. Koren *et al*. [14] suggested that the majority of bacterial genomes could be assembled into finished-grade quality, that is, without gaps, and with data derived from a single PacBio sequencing library per sample [14]. The combination of the longer reads, depth of coverage and random nature of sequencing errors facilitates *de novo* assemblies for microbial isolates [15,20,21]. The advantages of single-molecule sequencing have been discussed [22]. To date, relatively few genomes sequences have been determined exclusively using single-molecule technology and only a handful represent finished genomes [14,20,21,23-25].

In this study, a finished genome sequence for *Clostridium autoethanogenum* strain JA1-1 (DSM 10061) was generated using the latest PacBio RS II instrument. This represents one of the first *de novo* genomes finished into a single contiguous sequence using RS II data alone (that is, without addition of other next-generation sequence data or manual finishing steps). To offer insights into this technology, the PacBio assembly was compared to assemblies based on 454 GS FLX Titanium and Illumina MiSeq data and an earlier draft genome sequence of 100 contigs for this strain obtained from 454 GS FLX Titanium and Ion Torrent data [26].

*C. autoethanogenum* is an anaerobic, Gram-positive, mesophilic, acetogenic bacterium isolated using carbon monoxide (CO) [27]. Other substrates include the greenhouse gas CO_2_ plus H_2_, pyruvate, xylose, arabinose, fructose, rhamnose, and L-glutamate. There is significant biotechnological interest in this organism as well as other acetogenic bacteria for their ability to use gases containing CO, H_2_ and CO_2_ as the sole source of carbon and energy for the production of fuel and chemicals at scale. The ability to use these gases in fermentative processes enables acetogens to potentially provide a route to more sustainable fuel and chemical production from a range of feedstocks including biomass and municipal solid waste-derived syngas, reformed biogas and industrial waste gases derived, for example, from steel production facilities [28-33].

## Results and discussion

### Sequencing output and assembly statistics for *C. autoethanogenum* DSM 10061

Sequencing statistics show that for each platform a large number of raw reads were attained that resulted in high degrees of genome coverage (Table 1). Raw Illumina data were trimmed and filtered before assembly, but in the case of the 454 and PacBio assemblers raw instrument output files were used. Bruno-Barcena *et al*. used a combination of 454 GS FLX Titanium and Ion Torrent Personal Genome Machine (PGM) data to generate a genome reported as 4.5 Mb for *C. autoethanogenum* DSM 10061 [26]. The number of 454 reads (452,052) and genome coverage (39×) from the earlier study was similar to this one (Table 1), although addition of the PGM reads resulted in 905,738 raw reads being used to generate the preliminary assembly by Newbler (version 2.6). The record [GenBank: ASZX00000000.1] for strain DSM 10061 draft genome is reported as 4,323,309 bp.

**Table 1:** Sequencing statistics

	**Number of reads**	**Total bases**	**Mean read length (bp)**	**Longest read (bp)**	**Coverage (×)**
454-3 kb PE	511,515	202,048,425	395	945	46×
Illumina PE	3,689,644	553,446,600	151	151	127×
PacBio	122,933	782,530,012	6,366	26,777	179×

In this study, Newbler (version 2.8) was used to assemble new 454 paired-end reads from a 3-kb insert length library (Table 1) into a draft genome sequence that consisted of 32 contigs (Table 2). The lower number of contigs (32 versus 100) from the new 454-only assembly compared to the draft version [26] is likely due to differences in library types (paired-end versus shotgun) and software versions. Assembly of Illumina-only data was conducted using the SPAdes [34], Velvet [35], Abyss [36] and the CLC Genomics Workbench (CLC Bio) assemblers and the best results were obtained by the Velvet assembler (Table 2). Previously, we have assembled genome sequences for a range of bacteria using a combination of 454 and Illumina technologies, whereby initial Illumina consensus sequences were shredded into 1.5-kbp overlapped fake reads and assembled together with the 454 data [37-42]. The best genome assembly obtained for strain DSM 10061 using second generation sequencing technologies employed such a hybrid approach, which is reflected in the lowest number of contigs, the largest single contig and highest N50 value (Table 2). Preliminary studies using the *Clostridium ljungdahlii* DSM 13528 genome as a reference and a PCR/Sanger sequencing strategy showed contigs could be joined by such an approach (Additional file 1). As manual finishing is time consuming the potential of PacBio data to generate finished microbial genome sequences was assessed.

**Table 2:** Assembly statistics for strain DSM 10061

	**Contigs (number)**	**Largest contig (bp)**	**Contig N50 (bp)**	**Genome size (Mb)**	**Scaffolds**	**Largest scaffold (bp)**	**Scaffold N50 (bp)**	**Assembler**
454/Ion Torrent ^*^	100	436,795	115,901	4.32				Newbler 2.6
Illumina only	57	460,940	255,482	4.3	53	769,812	328,660	Velvet 1.2
454 only	32	134,546	330,116	4.3	13	1,137,876	898,466	Newbler 2.8
Illumina/ 454 Hybrid	22	1,137,625	687,076	4.3	13	1,137,625	899,926	Newbler 2.8
PacBio	1	4,352,205	4,352,267	4.3	1	4,352,267	4,352,267	SMRT 2.0

^*^Previously published as a 4.5-Mb draft genome [26], but present [GenBank: ASZX00000000.1] as 4,323,309 bp.

Remarkably, one PacBio library preparation and two single molecule real-time sequencing (SMRT) cells produced sufficient sequence such that it could be assembled into one contiguous DNA fragment that represented the DSM 10061 genome. The PacBio genome assembly is a similar size to the other assemblies (Tables 1 and 2) and genome completeness was confirmed by sequence wrap-around. This is one of the first *de novo* sequenced genomes we are aware of that has been closed without manual finishing or additional data, despite the complexity of the *C. autoethanogenum* genome.

A comparison of the 454/Illumina hybrid assembly to the PacBio assembly showed there were small regions of overlap in the hybrid assembly that weakly joined contigs, and were supported by PCR and Sanger data, but there was insufficient support for the Newbler software to join them (Additional file 1A). PCR and Sanger data joined small gaps between contigs (for example, see Additional file 1B) in line with predictions using *C. ljungdahlii* DSM 13528 as a reference but in other examples much larger products were obtained compared to the predicted PCR product sizes (Additional file 1C). Other challenges involved using a related but different species, or strain from manual finishing included instances of software not being able to design PCR primers, not obtaining PCR products, and instances of obtaining multiple PCR products of different sizes and/or DNA smears.

### Assembly quality assessments and comparisons

The complexity of the *C. autoethanogenum* DSM 10061 genome sequence was assessed and it is classified as a class III genome, according to previously described criteria for repeat sequence content and type [14]. Class III genomes are defined as containing repeats that can include rRNA gene operons, many mid-scale repeats, such as insertion sequences and simple sequence repeats, and large phage-mediated repeats, duplications, or large tandem arrays that are considerably larger than the rRNA gene operon.

PacBio sequencing technology has a high error rate, which has been reported as being approximately 18% [3]. Due to the random nature of the error [15], it is however, possible to get a highly accurate consensus sequence when there is high coverage [14,20,21]. For genomes such as *C. autoethanogenu*m with extreme guanine and cytosine (G + C) contents (31.1 mol% G + C content) and long homonucleotide stretches this provides an advantage over other sequencing technologies.

**Figure 1:**
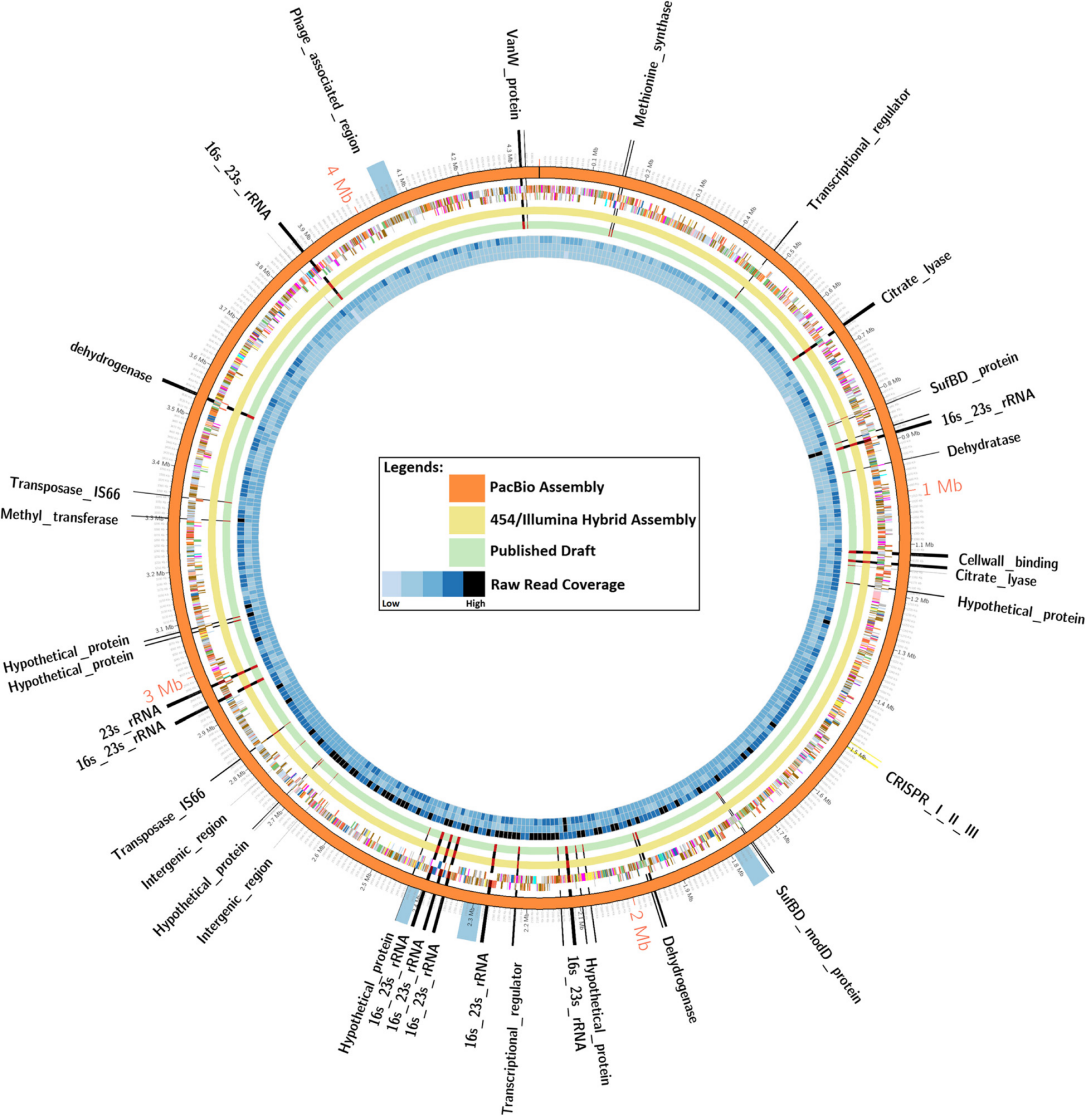
**Comparison of DSM10061 genome assemblies.** The orange colored ring represents the PacBio assembly. The next inner ring represents the genes encoded on positive and negative strands respectively and color coded by Clusters of Orthologous Groups (COG) categories. The 454/Illumina hybrid assembly and published draft assembly are represented as yellow and green circles, respectively. Next, three rings represent the raw-read coverage from PacBio, 454 and Illumina technology, respectively. The gaps in the 454/Illumina hybrid assembly and published draft assembly as compared to PacBio assembly are highlighted by red colors. The key genes in the gap regions are shown by black markers and intergenic regions are shown by gray markers. The phage region and CRISPR repeats are highlighted on PacBio assembly by blue and yellow color, respectively. Detail is provided in Table 3. CRISPR, clustered regularly interspaced short paloindromic repeats.

Beyond simple metrics, such as contig number, N50 and largest contig size, several bioinformatics approaches have been developed to assess assembly quality. The computing genome assembly likelihoods (CGAL) method is one recent approach that assesses uniformity of read coverage for assemblies and also evaluates the read errors, library insert size distribution and the degree of unassembled data [13]. At present, CGAL is only able to utilize Illumina reads for its assembly assessment and using Illumina reads it ranked the assemblies in the order of best to worst as Illumina only, Illumina/454 hybrid, 454, published draft, to PacBio, respectively (Additional file 2). The CGAL likelihood principle is based on the possibility that a read is produced from every single location in the assembly. Regions of repetitive DNA were to be sequenced by longer reads, which were at times not resolved by the Illumina reads (Figure 1) and this may have contributed to the lower CGAL scores for assemblies that contained longer reads and no Illumina data. QUAST [12], which used the PacBio assembly as the reference, ranked the Illumina/454 hybrid, 454, published draft, and Illumina only assemblies in the order of best to worst, respectively and details are provided (Additional file 3).

The tool, recognising errors in assemblies using paired reads (REAPR) for genome assembly evaluation [11] detected no collapsed repeats in the PacBio assembly and five in the hybrid assembly and four in each of the other assemblies (Additional file 4). The fragment coverage distribution (FCD) error detected by REAPR in PacBio assembly was at location 3872494 to 3873407 (913 bp). This region contains an rRNA gene operon and had very low Illumina coverage (40× as compared to the average of 127×). Hence, REAPR reported an error (based on Illumina reads only). Even 454 coverage was low in this region (19× as compared to average of 46×). However, there was 108× PacBio reads covering this (913 bp) region and for the first 392 bp there was also high-quality Sanger sequence support indicating it is unlikely that there is an issue for the PacBio assembly in this region. The hybrid and PacBio assemblies contained the fewest warnings (83 and 96, respectively), followed by the Illumina assembly (182) and then published draft assembly contained the most (190).

A multiple genome alignment was conducted by aligning contigs from the different assemblies to the PacBio reference assembly to identify conserved regions and to evaluate gaps in the different DSM 10061 assemblies. Regions with no or partial 454 or Illumina contig coverage predominantly contained predicted rRNA gene operons and other duplicated genes (Figure 1 and Table 3). While the draft genome sequence for strain DSM 10061 predicts one copy of the 16S rRNA gene [26], nine rRNA clusters were predicted using the DSM 10061 PacBio assembly, which is the same number of rRNA operons as in the closely related *C. ljungdahlii* DSM 13528 [28]. Based on findings in this study and earlier ones [1,3,14], the large number of DSM 10061 rRNA clusters and their repetitive nature confounded assembly of the shorter reads.

**Table 3:** Regions of low sequence-coverage

**Locus tag**	**Start**^**a**^	**End**^**a**^	**Product description**	**Pacbio coverage (×**^**b**^**)**	**454 Coverage (×)**	**Illumina coverage (×)**	**454 Hybrid contig coverage**^**c**^	**Draft assembly contig coverage**
CAETHG_0145	156117	156914	Methionine synthase	87	26	62	Complete	Partial
CAETHG_0152	161167	161292	Hypothetical protein	94	16	55	Complete	Partial
CAETHG_0153	161313	161963	Dihydropteroate synthase DHPS	93	22	46	Complete	Partial
CAETHG_0433	472649	474331	Transcriptional regulator, PucR family	110	25	57	Complete	Partial
CAETHG_0601	661798	663339	Citrate lyase, alpha subunit	109	25	64	Partial	Partial
**CAETHG_0602**	**663332**	**664234**	**Citrate lyase, beta subunit**	**111**	**29**	**65**	**None**	**None**
**CAETHG_0603**	**664234**	**664530**	**Citrate lyase acyl carrier protein**	**107**	**29**	**63**	**None**	**None**
**CAETHG_0604**	**664553**	**665587**	**Citrate lyase ligase**	**109**	**23**	**63**	**None**	**Partial**
**CAETHG_0605**	**665628**	**666806**	**Malic protein NAD-binding protein**	**101**	**27**	**69**	**None**	**None**
**Intergenic**	**827340**	**827520**	**NA**	**106**	**30**	**53**	**None**	**None**
CAETHG_0774	832108	833028	SufBD protein	109	23	65	Complete	Partial
CAETHG_0814	873533	874333	Hypothetical protein	106	23	69	Complete	None
CAETHG_0815	874375	874953	Hypothetical protein	102	23	55	Complete	None
**rRNA**	**885055**	**887942**	**23s_rRNA**	**87**	**77**	**147**	**None**	**None**
**rRNA**	**888206**	**889703**	**16s_rRNA**	**102**	**56**	**165**	**None**	**None**
CAETHG_0871	940541	941353	3-dehydroquinate dehydratase	109	27	59	Complete	Partial
**CAETHG_1038**	**1116305**	**1121431**	**Cell wall binding repeat 2-containing protein**	**127**	**27**	**69**	**Partial**	**None**
**CAETHG_1052**	**1136476**	**1138017**	**Citrate lyase, alpha subunit**	**107**	**22**	**53**	**Partial**	**None**
CAETHG_1053	1138010	1138912	Citrate lyase, beta subunit	106	29	75	Complete	None
CAETHG_1054	1138912	1139208	Citrate lyase acyl carrier protein	109	37	70	Complete	None
**CAETHG_1055**	**1139370**	**1140533**	**Malic protein NAD-binding protein**	**107**	**27**	**51**	**Partial**	**Partial**
Intergenic	1148600	1148780	NA	131	16	63	Complete	None
CAETHG_1100	1186843	1187643	Hypothetical protein	118	23	68	Complete	None
CAETHG_1101	1187685	1188263	Hypothetical protein	105	28	59	Complete	None
CAETHG_1630	1752229	1753149	SufBD protein	118	26	79	Complete	Partial
CAETHG_1634	1755642	1756505	modD protein	115	22	69	Complete	Partial
CAETHG_1708	1841018	1841572	Lumazine-binding	132	23	66	Complete	Complete
CAETHG_1816	1956238	1956534	Microcompartments protein	138	35	76	Complete	Partial
CAETHG_1817	1956609	1956899	Microcompartments protein	139	19	81	Complete	None
CAETHG_1818	1956948	1957598	Propanediol utilization protein	144	24	74	Complete	None
CAETHG_1819	1957600	1959153	Acetaldehyde dehydrogenase (acetylating)	153	25	67	Complete	None
CAETHG_1826	1963196	1964038	Ethanolamine utilization protein EutJ family protein	161	34	73	Complete	Partial
CAETHG_1827	1964020	1964790	Hypothetical protein	162	22	68	Complete	Partial
CAETHG_1949	2079078	2080271	Hypothetical protein	161	30	79	Complete	Partial
CAETHG_1963	2095013	2096206	Hypothetical protein	128	36	97	Complete	Partial
tRNA	2113813	2113886	tRNA_Met	128	15	61	None	Complete
**rRNA**	**2114155**	**2117042**	**23s_rRNA**	**122**	**81**	**161**	**None**	**None**
**rRNA**	**2117334**	**2118831**	**16s_rRNA**	**118**	**66**	**128**	**None**	**None**
tRNA	2135117	2135189	tRNA_Met	132	22	64	Complete	None
tRNA	2135201	2135286	tRNA_Leu	133	16	59	Complete	None
tRNA	2135301	2135374	tRNA_Met	133	17	57	Complete	None
tRNA	2135394	2136466	tRNA_Met	139	35	74	Complete	None
tRNA	2135478	2135563	tRNA_Leu	140	30	62	Complete	None
**CAETHG_2076**	**2220169**	**2221506**	**Sigma54 specific transcriptional regulator, Fis family**	**122**	**32**	**85**	**Partial**	**Partial**
**CAETHG_2077**	**2221658**	**2221885**	**Transcriptional regulator, Fis family**	**126**	**21**	**92**	**Partial**	**None**
**CAETHG_2078**	**2222014**	**2222994**	**Putative sigma54 specific transcriptional regulator**	**135**	**30**	**77**	**Partial**	**Partial**
**rRNA**	**2271738**	**2273235**	**16s_rRNA**	**165**	**10**	**26**	**None**	**None**
**rRNA**	**2273527**	**2276414**	**23s_rRNA**	**158**	**10**	**26**	**None**	**None**
tRNA	2276744	2276817	tRNA_Met	153	28	70	None	Complete
**rRNA**	**2355334**	**2356831**	**16s_rRNA**	**145**	**11**	**24**	**None**	**None**
**rRNA**	**2357123**	**2360010**	**23s_rRNA**	**136**	**13**	**23**	**None**	**None**
tRNA	2360340	2360412	tRNA_Lys	122	15	65	Complete	Partial
**rRNA**	**2372238**	**2373735**	**16s_rRNA**	**128**	**13**	**21**	**None**	**None**
**rRNA**	**2374027**	**2376914**	**23s_rRNA**	**126**	**14**	**19**	**None**	**None**
**rRNA**	**2392702**	**2394199**	**16s_rRNA**	**134**	**12**	**20**	**None**	**None**
**rRNA**	**2394596**	**2397483**	**23s_rRNA**	**142**	**11**	**21**	**None**	**None**
CAETHG_2238	2397706	2397882	Hypothetical protein	138	23	57	Partial	Complete
CAETHG_2268	2424703	2425503	Integrase catalytic region	115	26	61	Complete	None
CAETHG_2269	2425545	2426123	Hypothetical protein	124	26	56	Complete	None
Intergenic	2666300	2666515	NA	145	25	69	Complete	None
Intergenic	2710650	2710840	NA	124	36	71	Complete	None
CAETHG_2526	2714747	2715550	Hypothetical protein	133	28	74	Complete	Partial
Intergenic	2769840	2769880	NA	124	23	67	Complete	None
CAETHG_2620	2822788	2823741	Transposase IS66	124	30	59	Partial	Complete
**CAETHG_2621**	**2823723**	**2824328**	**Transposase IS66**	**127**	**30**	**52**	**Partial**	**Partial**
**rRNA**	**2935186**	**2936683**	**16s_rRNA**	**127**	**14**	**27**	**None**	**None**
**tRNA**	**2936973**	**2937045**	**tRNA_Ala**	**125**	**19**	**51**	**None**	**None**
**tRNA**	**2937053**	**2937126**	**tRNA_Ile**	**125**	**26**	**58**	**None**	**None**
**rRNA**	**2937443**	**2940330**	**23s_rRNA**	**117**	**14**	**28**	**None**	**None**
**rRNA**	**2966992**	**2968489**	**16s_rRNA**	**126**	**11**	**20**	**None**	**None**
**tRNA**	**2968779**	**2968851**	**tRNA_Ala**	**132**	**20**	**50**	**None**	**None**
**tRNA**	**2968859**	**2968932**	**tRNA_Ile**	**131**	**23**	**70**	**None**	**None**
**rRNA**	**2969222**	**2972109**	**23s_rRNA**	**128**	**10**	**19**	**None**	**None**
CAETHG_2843	3078642	3079445	Dihydropteroate synthase DHPS	152	30	66	Complete	Partial
CAETHG_2844	3079499	3080131	Hypothetical protein	148	32	71	Complete	Partial
CAETHG_2848	3085939	3086742	Dihydropteroate synthase DHPS	146	27	66	Complete	Partial
CAETHG_2849	3086796	3087428	Hypothetical protein	139	31	75	Complete	Partial
CAETHG_3037	3301321	3302088	MCP methyltransferase, CheR-type	149	23	65	Complete	Partial
CAETHG_3075	3342748	3343524	Transposase IS66	112	39	74	Complete	Partial
CAETHG_3281	3537107	3537880	Hypothetical protein	109	27	55	Complete	Partial
CAETHG_3282	3537862	3538704	Ethanolamine utilization protein	107	30	62	Complete	None
CAETHG_3283	3538721	3539026	Microcompartments protein	103	20	65	Complete	None
CAETHG_3284	3539020	3539286	Ethanolamine utilization protein EutN/carboxysome structural protein Ccml	106	25	55	Complete	None
CAETHG_3285	3539304	3539975	Ethanolamine utilization EutQ family protein	110	29	63	Complete	None
CAETHG_3286	3540008	3540784	Microcompartments protein	106	30	61	Complete	None
CAETHG_3287	3540833	3542350	Acetaldehyde dehydrogenase (acetylating)	111	27	61	Complete	Partial
Intergenic	3848150	3848350	NA	126	34	39	Complete	None
**rRNA**	**3872016**	**3873511**	**16s_rRNA**	**98**	**10**	**18**	**None**	**None**
**rRNA**	**3873937**	**3876824**	**23s_rRNA**	**107**	**14**	**21**	**None**	**None**
CAETHG_4028	4315106	4316413	VanW family protein	98	24	66	Complete	Partial
CAETHG_4029	4316730	4319132	Collagen triple helix repeat-containing protein	94	13	38	Complete	Partial
CAETHG_4035	4325792	4326292	VanW family protein	78	21	54	Complete	Partial

^**a**^The genomic regions which were not assembled in 454/Draft assembly are listed above; ^**b**^the ‘x’ coverage defines the raw-read coverage averaged over given coordinates; ^**c**^‘Complete/partial’ contig coverage defines whether the region was completely/partially assembled while ‘None’ defines that this region is missing in the respective assembly. Missing regions in either 454/Draft assembly are shown in bold.

The latest PacBio RS II SMRT cells are designed to select for larger read-lengths when long insert libraries (10 to 20 kb) are being prepared, however, preferential loading of smaller fragments can still occur and this limits sequence output. In this study, smaller fragments were removed from the PacBio library by size exclusion leading to longer read-lengths and greater amounts of sequence data than otherwise might have been attained. The long reads produced by the new PacBio RS II system, combined with sequence depth meant that the principal regions of complexity could be resolved using one library preparation and two SMRT cells to generate a complete genome sequence. The application of long, single-molecule sequencing data will lead to a greater number of finished genomes and quality improvements in microbial genome databases [14], however the application of the newest version of this technology requires more evaluation before its full potential can be assessed for complex genomes.

### General features of the *C. autoethanogenum* genome, its metabolism and comparison to *C. ljungdahlii*

The finished genome of *C. autoethanogenum* DSM 10061 consists of one chromosome of 4,352,205 bp in size with a G + C content of 31.1 mol% and consists of 89 RNA genes (Additional file 5). Of the 4,161 genes predicted for this strain, 4,042 are protein-coding genes (CDSs) and 18 are pseudogenes. The distribution of genes into COG functional categories is presented (Additional file 6). The previously published draft DSM 10061 genome annotation included 4,135 predicted coding sequences [26] and the related finished *C. ljungdahlii* DSM 13528 genome which is 277,860 bp larger in size contained 4,184 protein coding genes [28]. Predicted gene content differences reflect the use of different gene-calling algorithms, that draft sequences can split genes in two, and genotypic differences. The methodology, accuracy, and specificity of the Prodigal gene prediction algorithm used in this study has been described previously [43].

Phenotypic and metabolic differences have been reported for *C. autoethanogenum* and *C. ljungdahlii*[27,44-47]. The two are indistinguishable at the 16S rRNA gene level [48] and have high scores for similarity based on *in silico* average nucleotide identity comparisons across the genomes (0.9977 ANIb) [26]. To evaluate potential coding sequence differences between the two organisms, OrthoMCL [49], a genome-scale algorithm for grouping orthologous protein sequences, was used to compare all the *C. autoethanogenum* proteins to those in *C. ljungdahlii* and for the reciprocal evaluation (Additional file 7). A general description for all OrthoMCL proteins is provided (Additional file 7: Table S1). Putative paralogs were identified (1_taxa tab in Additional file 7) along with putative orthologs (2_taxa file tab in Additional file 7). Proteins without orthologs or paralogs were identified using the default settings (*C. autoethanogenum* unique or *C. ljungdahlii* unique tabs in Additional file 7). This analysis revealed that over 10% of the proteome is unique to each bacterium when comparing *C. autoethanogenum* (427 proteins out of 4,134) and *C. ljungdahlii* (447 out of 4,198). The 427 proteins with unique genes to DSM 10061 (as listed by OrthoMCL) were searched against the entire *C. ljungdahlii* proteome using BLASTP and an e-value similarity criteria of 1e-5 to identify proteins with truly unique function and no homolog, which reduced the number of dissimilar or unique proteins to 221 (BLASTP analysis tab in Additional file 7). From the proteins identified as unique to each bacterium, the majority were proteins with hypothetical functions or proteins related to particular phage, transposon or CRISPR sequences, but proteins with key functions in the metabolism were also identified that could explain different phenotypes. These differences are discussed below.

**Figure 2:**
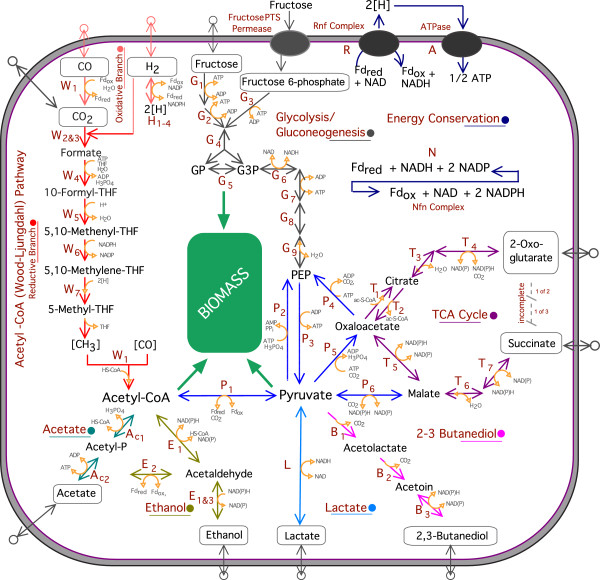
**Inferred metabolism of *C. autoethanogenum*.** Capital letters in brown denote enzymes. ATP, adenosine triphosphate, ADP, adenosine diphosphate; BDO, 2,3-butanediol; CO, carbon monoxide; CO2, carbon dioxide; FAD, flavin adenine dinucleotide; FADH2 FD_red, ferredoxin (reduced); FD_ox, ferredoxin (oxidized); G3P, 3-phosphoglycerate; GP, glycerone-phosphate; H3PO4, phosphate; NAD, nicotinamide adenine dinucleotide (oxidized); NADH, nicotinamide adenine dinucleotide (reduced); NADP, nicotinamide adenine dinucleotide phosphate (oxidized); NADPH, nicotinamide adenine dinucleotide phosphate (reduced); TCA, tricarboxcylic acid cycle. Note that reaction directionality has not been rigorously determined; in general, directionality is as reported in KEGG reactions. **Acetyl-CoA (Wood-Ljungdahl) pathway – Reductive branch.** W1 Bifunctional CO dehydrogenase/Acetyl-CoA synthase (CODH/ACS) CAETHG_1620-21, 1608-11, W2 Seleno formate dehydrogenase (Fdh) CAETHG_0084, 2789, W3 Non-seleno formate dehydrogenase (Fdh) CAETHG_2988, W_4_ Formyl-THF ligase (Fhs) CAETHG_1618, W_5_ Methenyl-THF cyclohydrolase (FchA) CAETHG_1617, W_6_ Methylene-THF dehydrogenase (FolD) CAETHG_1616, W_7_ Methylene-THF reductase (MetF) CAETHG_1614-15. **Acetyl-CoA (Wood-Ljungdahl) pathway – Oxidative branch.** C Monofunctional CO dehydrogenase CAETHG_3899, 3005, H_1_ Electron-bifurcating [FeFe] Hydrogenase (HytCBDE1AE2) CAETHG_2798, H_2_ Other [FeFe] hydrogenases (Hyd) CAETHG_0110, 0120, 1576, 3569, 3841, H_3_ [NiFe] hydrogenase (Hyd) CAETHG_0862, H_4_ Hydrogenase maturation factor (HypEDCF) CAETHG_0368-0371. **Energy conservation.** A F_1_F_O_ ATPase (AtpIBEFHAGDC) CAETHG_2342-50, N Electron-bifurcating NADH-dependent Fd:NADP oxidoreductase (Nfn) CAETHG_1580, R Rnf complex (RnfCDGEAB) CAETHG_3227-32. **Acetate fermentation pathway.** Ac_1_ Phosphotransacetylase (Pta) CAETHG_3358, Ac_2_ Acetate kinase (Ack) CAETHG_3359. **Ethanol fermentation pathway.** E_1_ Bifunctional aldehyde/alcohol dehydrogenase (AdhE) CAETHG_3747, 3748, E_2_ Aldehyde:Fd oxidoreductase (AOR) CAETHG_0092, 0102, E_3_ Additional alcohol dehydrogenases (Adh) CAETHG_0555. **2,3-butanediol fermentation pathway.** B_1_ Acetolactate synthase (AlsS) CAETHG_0124-25, 0406, 1740, B_2_ Acetolactate decarboxylase (BudA) CAETHG_2932, B_3_ 2,3-butanediol dehydrogenase (Bdh) CAETHG_0385, **Lactate fermentation pathway.** L Lactate dehydrogenase (Ldh) CAETHG_1147. **Central pyruvate metabolism.** P_1_ Pyruvate:ferredoxin oxidoreductase (PFOR) CAETHG_0928, 3029, P_2_ Pyruvate, phosphate dikinase (PPDK) CAETHG_2055, 2909, P_3_ Pyruvate kinase (Pk) CAETHG_2440-41, P_4_ Pyruvate carboxylase (Pyc) CAETHG_1594, P_5_ PEP carboxykinase (PEPCK) CAETHG_2721, P_5_ Malic enzyme CAETHG_0605, 1055. **Incomplete TCA cycle.** T_1_ Citrate synthase CAETHG _2751, T_2_ Citrate lyase CAETHG_1052-54, 1898–1901, 2480-83, T_3_ Aconitase (Aco) CAETHG_1051, 2752, T_4_ Isocitrate dehydrogenase (Idh) CAETHG_2753, T_5_ Malate dehydrogenase (Mdh) CAETHG_1702, 2478, 2689, T_6_ Fumarase CAETHG_1902-03, 2062, 2479, T_7_ Fumarate reductase CAETHG_0344, 1032, 2961. **Glycolysis/Gluconeogenesis.** PTS Fructose phosphotransferase system (PTS) CAETHG_0142, 0676-73, G_1_ Fructokinase (Fk) /Fructose-6-phosphate isomerase CAETHG_0166, 0156, G_2_ 1-phosphofructokinase (Pfk1) CAETHG_0143, G_3_ 6-phosphofructokinase (Pfk6) CAETHG_648, 2439, G_4_ Fructose bisphosphate aldolase (Aldo) CAETHG_2382, G_5_ Triose-phosphate isomerase (Tpi) CAETHG_1758, G_6_ Glyceraldehyde-3-phosphate dehydrogenase (GAPDH) CAETHG_1760, 3424, G_7_ Phosphoglycerate kinase (Pgk) CAETHG_1759, G_8_ Phosphoglycerate mutase (Pgm) CAETHG_712, 1757, G_9_ Enolase phosphopyruvate hydratase (Eno) CAETHG_1756.

The Wood-Ljungdahl pathway (Figure 2) plays a key role in the acetogenic metabolism by allowing the formation of acetyl-CoA from CO or CO_2_, and thus, is essential for autotrophic growth. Under heterotrophic growth conditions it permits utilization of produced CO_2_ and reducing equivalents generated during glycolysis to form an additional molecule of acetyl-CoA [50]. The genes encoding for the enzymes of the Wood-Ljungdahl pathway are co-localized in one large cluster (CAETHG_ 1606–1621). The same organization is also found in other acetogens such as *C. ljungdahlii*[28], *C. ragsdalei*[28] or *C. difficile*[51], but significant differences at the sequence level are present as described earlier [28,51]. This cluster also includes the genes for the bifunctional carbon monoxide dehydrogenase/acetyl-CoA synthase (CODH/ACS) enzyme complex, the key enzyme in the Wood-Ljungdahl pathway. As in *C. ljungdahlii*, two additional monofunctional carbon monoxide dehydrogenases (CAETHG_3005 and CAETHG_3899) are encoded in the genome of *C. autoethanogenum* that may also be involved in utilization of CO and CO_2_. Although CO can be both a carbon and an energy source for the bacteria, CO_2_ can only be used as a carbon source. Additional energy can be generated from hydrogen, via hydrogenase enzymes. The genome of *C. autoethanogenum* encodes for six hydrogenases, one (NiFe) hydrogenase and five (FeFe) hydrogenases. Interestingly, *C. ljungdahlii* only has five hydrogenases, lacking one of the iron-only hydrogenases that are present in *C. autoethanogenum*. The genes for this unique (FeFe) hydrogenase are in an operon with two genes for NuoF-like oxidoreductases (CAETHG_1575-78). The presence of an additional hydrogenase enzyme complex could represent a significant advantage for *C. autoethanogenum* during autotrophic growth on CO, CO_2_ and H_2_ containing gases. Preliminary RNA-Seq experiments show that this cluster is highly expressed under such conditions, underlining the importance of this enzyme (Additional file 8). Of the other *C. autoethanogenum* hydrogenases, a second (FeFe) hydrogenase gene cluster was also found to be highly expressed. This nicotinamide adenine dinucleotide phosphate-oxidase (NADPH)-specific electron-bifurcating Hyt hydrogenase was recently characterized and found to form a functional complex with a formate dehydrogenase [52]. Formate dehydrogenase activates CO_2_ to formate in the Wood-Ljungdahl pathway and additional formate dehydrogenase genes are present in the *C. autoethanogenum* genome (Figure 2). *C. autoethanogenum* also has a predicted formate transporter (CAETHG_1601) that is not present in *C. ljungdahlii*.

During autotrophic growth, all biomass and products must be derived of acetyl-CoA from the Wood-Ljungdahl pathway (Figure 2). Fatty acid biosynthesis starts directly from acetyl-CoA, whereas production of nucleic acids, amino acids, vitamins, cofactors and secondary metabolites proceed via pyruvate and gluconeogenesis or the TCA cycle. The *C. autoethanogenum* genome encodes for two pyruvate:ferredoxin oxidoreductases (PFOR) that catalyze the conversion of acetyl-CoA into pyruvate. *C. autoethanogenum* has a pyruvate, phosphate dikinase (PPDK), but interestingly no phophoenolpyruvate synthase (PPSA) and *C. ljungdahlii* contains two such enzymes (CLJU_c14340 and CLJU_c38600). The rest of gluconeogenesis is similar in both organisms. *C. autoethanogenum* has an incomplete TCA cycle to succinate and 3-oxogluterate (Figure 2). *C. autoethanogenum* products such as 2,3-butanediol and lactate are also derived from pyruvate [28], whereas ethanol is produced from acetyl-CoA via acetaldehyde, either directly via bifunctional aldehyde/alcohol dehydrogenases or via acetate using phosphotransacetylase and acetate kinase and an aldehyde:ferredoxin oxidoreductase (Figure 2). Several additional alcohol dehydrogenases are present in the genome of *C. autoethanogenum*.

Heterotrophic growth on a range of other products such as a range of C5 and C6 sugars has been described for *C. autoethanogenum*[27]. A PTS system and other respective genes could be identified in the genome (Figure 2). In contrast to *C. ljungdahlii*, some extra genes involved in mannose metabolism are present in *C. autoethanogenum* as well as genes for aromatic compound degradation. *C. autoethanogenum* also has an additional predicted nitrate reductase (CAETHG_0085) and both organisms differ in some of their transport systems.

Other differences between *C. autoethanogenum* and *C. ljungdahlii* include variations in the sporulation program, with several unique predicted sporulation proteins and regulators present in *C. autoethanogenum* strain DSM 10061, and different defense systems, such as restriction/methylation systems and a CRISPR system that is present in *C. autoethanogenum* but not in *C. ljungdahlii*. Insertion sequence (IS) elements, are usually unique to a strain, and one is found in *C. autoethanogenum* between 4,345,780 and 4,347,448 bp that is 100% identical to one in *C. ljungdahlii. C. autoethanogenum* DSM 10061 was enriched from rabbit feces in Belgium [27] and *C. ljungdahlii* DSM 13528 was isolated in the US from chicken yard waste [47]. Despite the geographical separation of the isolates, the overall degree of similarity between *C. ljungdahlii* and *C. autoethanogenum* suggests a common ancestor.

### *C. autoethanogenum* CRISPR system

CRISPR are prokaryotic DNA loci that carry the memory of past bacterial infections of phages and plasmids to provide immunity against mobile genetic elements [53,54]. In the last decade, several studies have unraveled CRISPR defense molecular details and mechanisms of action [53,55,56]. Briefly, CRISPR loci are composed of arrays of 24 to 47 bp partially palindromic, highly conserved repeats separated by variable spacers specific to the infecting DNA. CRISPR-associated (*cas*) genes are involved in spacer acquisition, expression and interference to phage or plasmid. *cas* gene operons are classified into three types and several subtypes, and can target either DNA or RNA, or both [53]. CRISPR and *cas* gene operons are proposed to be transferred between distinctly related strains by horizontal gene transfer and/or by transposons [57], and the latter can be identified by the presence of insertion elements and transposase/mutase in its vicinity. Thus, CRISPR appear to be dynamic heritable defense systems in bacteria against plasmids and phages that are ever fast-evolving and play important roles in the co-evolution of both bacteria and phages.

The genome of *C. autoethanogenum* is found to contain eight *cas* genes of Type-I B, all predicted to be in one operon on the antisense strand with a predicted transcription terminator at the end of the *cas2* gene, and it is flanked by three CRISPR arrays (Table 4, Additional files 9 and 10) with a total of 93 30-bp-repeats (consensus 5′-GTTGAACCTCAACATGAGATGTATTTAAAT-3′) and 90 spacers of 35 to 38 bp. All three CRISPR arrays are preceded by a 177-bp-leader sequence, which is essential for array transcription and a fragment of the leader sequence is co-transcribed with the array [58,59]. The three putative *C. autoethanogenum* CRISPR arrays leader sequences share 82 to 91% sequence similarity between them (Additional file 9). Interestingly, 10 kb downstream of the three CRISPR arrays, an incomplete leader sequence of 65 bp was found that has high sequence identity to the other leaders in close proximity (100 bp) to an imperfect CRISPR repeat (5′-GTTGAACCTtAACATGAGATGTAaaggtAa-3′). In addition to the three CRISPR arrays flanking the *cas* genes, a putative extra CRISPR array was identified in the genome, consisting of three 55-bp-repeats and two 16-bp-spacer (Additional file 10).

**Table 4:** **Overview of CRISPR systems, plasmids and prophages in fuel-producing *Clostridium *** **species**

**Category**	**Organism**	**Genome size (Mb)**	**Status**	**Plasmid reference number**		**Plasmid(s) size (kb)**	Prophages (number)^*^	**Prophage(s) size (kb)**	CRISPR arrays (number)^*^	**CRISPR repeats (number)**
Solventogenic (ABE) Clostridia	*C. acetobutylicum* ATCC824	3.94	complete	[60]	1	192	3	191	-	-
	*C. acetobutylicum* EA2018	3.94	complete	[61]	1	192	3	191	-	-
	*C. acetobutylicum* DSM1731	3.94	complete	[62]	2	201	3	191	-	-
	*C. beijerinckii* NCIMB8052	6.00	complete	-	-	-	4	106	-	-
	*C. saccharobutylicum* DSM13864	5.10	complete	[63]	-	-	5	133	4	55
	*C. saccharoperbutylacetonicum* N1-4	6.53	complete	[64]	1	136	6	161	4	67
Cellulolytic Clostridia	*C. cellulolyticum* H10	4.07	complete	-	-	-	8	210	3	23
	*C. cellulovoran*s 743B	5.26	complete	[65]	-	-	5	179	3	44
	*C. thermocellum* ATCC27405	3.84	complete	[66]	-	-	5	222	5	442
	*C. thermocellum* DSM1313	3.56	complete	[67]	-	-	1	26	5	189
	*C. phytofermentans* ISdg	4.85	complete	-	-	-	1	28	-	-
Acetogenic Clostridia	*C. autoethanogenum* DSM10061	4.35	complete	this study	-	-	4	115	3	95
	*C. ljungdhalii* DSM13528	4.63	complete	[28]	-	-	6	248	-	-
	*C. carboxidivorans* P7	5.59	251 contigs	-	1	20	1	19	-	-
		4.40	69 contigs	[68]	1	20	-	-	-	-

^*^Please refer to Additional file 10: Table S9 for details.

Expression of *cas* genes and CRISPR arrays along with their leader sequence were studied by Reverse Transcriptase PCR (RT-PCR) and RNA-Seq during logarithmic growth under autotrophic conditions. PCR amplification of fragments of expected sizes were observed only with cDNA template and not with RNA, showing the absence of genomic DNA contamination in RNA preparations (Additional file 9). All eight predicted *cas* genes appear to be co-expressed and from a single operon. Expression of spacers distal to the leader sequences in all three arrays was also assessed. Based on sequence similarity between the three leader sequences, a common reverse primer was designed to align to the conserved region in the leader sequences of all three arrays and forward primers aligning specifically to spacers proximal to the leader sequence in each array (Additional file 9). Both the leader proximal and distal spacers of array 2 were found to be expressed, whereas expression was detected only for spacers proximal to the leader sequence in array 3 and no expression was detected from array 1 or the identified extra leader. Preliminary RNA-Seq data showed expression of all CRISPR RNAs (crRNAs), with different abundances. A few transcripts corresponding to the leader region of the three CRISPR arrays were also detected. Similar to the *cas* gene operon, the three CRISPR arrays including their leader regions were transcribed from the antisense strand. The three CRISPR arrays were found to be constitutively transcribed and processed into crRNAs of varying lengths (Additional file 8). However, the processed crRNAs appeared to have a well-defined eight-nucleotide 5′ handle, 5′-ATTTAAAT-3′, originating from the repeat region followed by the spacer sequence (Additional file 8). The 3′ end of these processed crRNAs had varying tags (Additional file 8). Based on these findings, a scheme for CRISPR processing in *C. autoethanogenum* is proposed (Additional file 8). Similar processing of crRNA was observed across the three samples collected at different time points.

CRISPR spacer sequences in *C. autoethanogenum* were analyzed to identify potential target DNA sequence. A BLAST search did not result in high identity hits within the National Center for Biotechnology Information (NCBI) database or against its own genome. A comparison of regions of DNA from putative *C. autoethanogenum* processing crRNAs from all three arrays identified the sequence 5′-ATTTAAAT-3′ (Additional file 9), which is similar to sequence*s* from *Clostridium thermocellum*[69], *Methanococcus maripaludis*[69], *Escherichia coli*[70] and *Pyrococcus furiosus*[71], which also have type-IB CRISPR systems. In these organisms the processing of crRNA is mediated by the *cas6* gene, which is also found in *C. autoethanogenum*. Unlike in *C. thermocellum*[69], *M. maripaludis*[69], *Sulfolobus acidocaldarius*[72] and *P. furiosus*[73], *C. autoethanogenum* crRNAs were transcribed only from the antisense strand and no anti-crRNA transcripts originating from the complementary strand were detected.

### Identification and classification of CRISPR systems in industrial relevant clostridia

The presence of a CRISPR system in *C. autoethanogenum* compared to *C. ljungdahlii* could provide an advantage in industrial fermentations. The *C. autoethanogenum* CRISPR system was compared to those from other industrial relevant Clostridia strains to better understand their characteristics and their potential physiological and applied roles. In particular, the Clostridial ABE fermentation process has a history of phage infections [74]. CRISPR systems from 14 *Clostridium* species were examined for the first time including those used in ABE fermentation processes: *C. acetobutylicum, C. beijerinckii*, *C. saccharobutylicum* and *C. saccharoperbutylicum*, cellulose degrading *C. thermocellum, C. cellulolyticum, C. cellulovorans,* and *C. phytofermentans*, and the acetogens *C. autoethanogenum, C. ljungdahlii* and *C. carboxidivorans*. CRISPR elements were identified only in 8 of the 14 *Clostridium* species analyzed by PILER [75] and CRISPRdb [76]. All of the loci were found on chromosomes and none on any plasmids or megaplasmids (Table 4). From the ABE fermentation-Clostridia examined, only *C. saccharobutylicum* DSM13864 has a CRISPR system, but not several strains of the more commonly used *C. acetobutylicum, C. beijerinckii* and *C. saccharobutylicum*. This may be one of the reasons why the ABE fermentation process was historically found to be prone to phage infections [74]. From the three acetogenic strains investigated only *C. autoethanogenum* had a CRISPR system, whereas all analyzed cellulolytic Clostridia, but *C. phytofermentans* contain CRISPR systems.

In all *Clostridium* species that harbor CRISPR arrays, *cas* genes were identified. *C. cellulolyticum* and *C. thermocellum* had two and four different *cas* operons, respectively (Additional files 10 and 11). These *cas* operons were classified based on a recently proposed classification system [53] and their target molecule(s) inferred. A phylogenetic analysis of *cas1* genes was performed and compared to the 16S rRNA phylogeny (Additional file 12). In *C. cellulolyticum*, arrays 1 and 2 are associated with the Type I-C *cas* system and Adb with the Type II *cas* system. The two arrays are separated by a transposase and mutase genes (Additional file 11). *C. cellulolyticum* has two different sets of *cas* genes, both of which appear to target DNA. The *C. cellulovorans cas* operon could not be classified according by these criteria, nor could the target of its *cas* genes be inferred. *C. thermocellum* appears to have a Type III *cas* genes system (Additional file 11). The Type III *cas* system contains more than one type of *cas* gene operon belonging to either Type I or II or the repeat-associated mysterious proteins (RAMP) module operon and are predicted to target both DNA and RNA [53]. The arrays 3 and 4 in *C. thermocellum* are associated with the Type I-B *cas* system [69] and arrays 5 and 6 to a *cas* system similar to Type I-B but interrupted by insertion of multiple other genes that separate *cas1*, *cas2, cas4* genes (possibly involved in spacer acquisition) from *cas3, 5, 7* and *8b* (predicted to be involved in DNA interference) (Additional file 11). The array 1 is not associated with any *cas* gene cassette and is flanked by a integrase and mutase genes at the 3′ end. Apart from these two *cas* systems, *C. thermocellum* also has a RAMP module gene cassette that may be involved in RNA interference. This cassette is not associated with any array and could be acting in *trans*. As in a few lactic acid bacteria [57], integrase and mutase genes were frequently found near the *cas* gene cassette, particularly flanking the Type 1 *cas* gene cassette found in *C. cellulovorans*, *C. thermocellum* and *C. autoethanogenum*, suggesting possible horizontal gene transfer. These genes were also found next to array-1 in *C. thermocellum*.

The *C. autoethanogenum* CRISPR repeat DNA was not found in any of the other *Clostridium* species included in this study. A search for organisms with repeats similar to *C. autoethanogenum* in the CRISPRdb database [75] resulted in *Clostridium novyi*, *Eubacterium limosum*, along with a few *Clostridium botulinum* substrains. A comparison of the repeat sequences showed very high sequence similarity (Additional file 12). The *cas* genes operon in *C. autoethanogenum*, *C. novyi* and *E. limosum* were all of Type-I B, whereas the *C. botulinum* substrains had different sets of *cas* genes. The *cas* gene operon architecture, the arrangement of arrays on the chromosome and the presence of two hypothetical genes separating arrays 2 and 3 in *C. autoethanogenum* and *C. novyi* are strikingly alike, suggesting a common lineage of these two CRISPR-*cas* systems. This observation was further strengthened by the phylogenetic classification placing *C. autoethanogenum cas1* gene together with *cas1* genes from *C. novyi* and *E. limosum* and apart from the other *Clostridium* species (Additional file 12). Even though the repeat and the *cas* genes operon in *C. autoethanogenum*, *C. novyi* and *E. limosum* are largely identical, no similarity was found between the spacers.

### Comparison of strains with/without the CRISPR system to plasmid and prophage content

Correlation between the presence of CRISPR and the occurrence of prophages or plasmids has been reported [77]. To assess the 14 *Clostridium* species for this correlation their genome sequences were analyzed for presence of potential prophage regions. In *C. autoethanogenum*, four putative prophages were identified, an incomplete prophage similar to a Singapore grouper iridovirus, an intact prophage similar to a *Geobacillus* E2 virus and two intact prophages inserted into tRNAs (Additional file 10). One prophage was identified a Trp-tRNA and the other in an Arg-tRNA. The latter is in almost identical form also present in *C. ljungdahlii*, suggesting a shared lineage*.* Prophage regions were detected in all species irrespective of the presence of CRISPR modules (Table 4 and Additional file 10). Although there seems to be no general trend and it cannot be determined whether a prophage infection occurred before or after a CRISPR system was acquired, in a few cases bacteria that lacked CRISPR systems appeared to have more abundant prophage sequences.

When looking for plasmid content, only one out of seven strains containing CRISPR systems was found to contain a plasmid. Likewise, only one out of five plasmid-carrying strains contained a CRISPR system. CRISPR-mediated immunity has been shown experimentally to block conjugative plasmid acquisition [78], although the role of CRISPR in driving plasmid and phage evolution for industrially relevant Clostridia and other microorganisms remains to be fully elucidated.

## Conclusions

A comparative genomic analysis revealed short-read technologies were unable to overcome *C. autoethanogenum* DSM 10061 repeat regions largely associated with nine copies of the rRNA gene operons. A previous study suggested that long single-molecule reads are sufficient to assemble most known microbial genomes based on a bioinformatics analysis of 2,267 complete genomes for bacteria and archaea and sequencing results for six bacteria [14]. The genome sequence of *C. autoethanogenum* DSM 10061 is classified as within the most complex class of bacterial genomes and a complete genome sequence was generated for it using long single-molecule reads and without the need for manual finishing. The relatively low cost to generate the PacBio data (approximately US$1,500) and the outcome of this study support the assertion this technology will be valuable in future studies where a complete genome sequence is important and for complex genomes that contain large repeat elements.

Clostridia are known for their substrate and metabolic flexibility, which makes them attractive biocatalysts for biofuel and biorefinery applications [79]. Acetogenic Clostridia, such as *C. autoethanogenum*, are of interest due to their ability to ferment abundant syngas or waste gases to useful products [29]. The *C. autoethanogenum* genome sequence will facilitate strain development for biofuels and biochemicals production and comparative genomics in the future. A comparison between *C. autoethanogenum* and *C. ljungdahlii* identified distinct differences, notably the presence of a CRISPR system, an additional *C. autoethanogenum* hydrogenase, and several differences in central metabolism, although the two bacteria likely descend from a common ancestor. Comparative genomic analysis and characterization of CRISPR, plasmid content and prophage among Clostridia with biotechnological interest was performed. Notably, the classic ABE fermentation strains *C. acetobutylicum* and *C. beijeinckii* are reported to be prone to bacteriophage infections [63] and all lack a CRISPR system and only one of the analyzed 14 strains contain both a plasmid and a CRISPR system. From the acetogenic *Clostridium* strains sequenced to date, only *C. autoethanogenum* possesses a CRISPR system. Further consideration of Clostridia CRISPR systems may be informative for bioprocess development strategies and for ecological studies.

## Methods

### DNA sequence data generation

*C. autoethanogenum* strain JA1-1 was obtained from the Deutsche Sammlung von Mikroorganismen und Zellkulturen (DSMZ) culture collection (DSM 10061). *C. autoethanogenum* strain JA1-1 was cultured in PETC medium as described [28]. Single colony was purified and 16S rDNA sequence confirmed before genomic DNA was prepared. High molecular weight genomic DNA was prepared as described earlier [28], quantified with a NanoDrop ND-1000 spectrophotometer (NanoDrop Technologies, Wilmington, DE, USA) and quality was assessed with Agilent Bioanalyzer (Agilent, Santa Clara, CA, USA).

Pyrosequencing was conducted using the Roche 454 GS FLX System (Roche 454 Life Sciences, Branford, CT, USA) with the method of paired-end DNA library preparation and average insert sizes in the 3-kb range and Titanium chemistry, according to the manufacturer’s instructions as described previously [38,80]. Sequence data were also generated using a MiSeq instrument (Illumina, San Diego, CA, USA) [16] and a paired-end approach with an approximate insert library size of 500 bp and read lengths of 151 bp, as described previously [81] and according to the manufacturer’s instructions. DNA for PacBio sequencing was sheared with G-tubes (Covaris, Inc., Woburn, MA, USA), targeting 20-kb fragments. PacBio libraries were prepared with the DNA Template Prep Kit 2.0 (Pacific Biosciences, Menlo Park, CA, USA) and library fragments above 4 kb were isolated using the Blue Pippin system (Sage Science, Inc., Beverly, MA, USA). The average PacBio library insert size (including adapters) was approximately 19 kb and samples were sequenced using Magbead loading, C2 chemistry, Polymerase version P4, and software version 2.02. Raw next-generation sequence data available through the NCBI SRA database [SRX352885; SRX352888; SRP030033]. PCR and Sanger sequencing were conducted using standard approaches as described previously [82] and primer sequences are described (Additional file 1).

### Sequence data trimming, filtering, annotation and assembly

The CLC Genomics Workbench (version 6.0.2) (CLC bio, Cambridge, MA, USA) was used to trim and filter Illumina reads for quality sequence data and the subsequent Illumina assembly. The Newbler application (version 2.8) in the 454 GS FLX software package (Roche 454 Life Sciences) was used to assemble reads generated from the GS FLX instrument and in combination with reads from the Illumina instrument, as described previously [38]. The consensus Illumina sequences were processed before inputting into the Newbler assembler by generating 1.5-kb overlapping fake reads using the fb_dice.pl script, which is part of the FragBlast module (http://www.clarkfrancis.com/codes/fb_dice.pl). The PacBio reads were assembled through SMRTanalysis v 2.0 (Pacific Biosciences) using the HGAP protocol [20]. The DSM 10061 PacBio assembly was annotated using the Prodigal gene-calling algorithm [43] and deposited in the NCBI database [GenBank: CP006763].

### Assessment of genome assembly quality

The *in silico* evaluation of genome assemblies was performed using CGAL (version 0.9.6) [13], REAPR (version 1.0.16) [11], QUAST (version 2.2) [12] and Circos [83]. The genomic repeats were identified using Nucmer [84]; genome complexity was determined based on count and length of the repeats as suggested earlier [14]. Gaps in the 454/Illumina hybrid and published draft assemblies were determined by performing multiple genome alignment through Mauve (version 2.3.1) [85] with PacBio assembly used as reference genome. The order of contigs in 454/Illumina hybrid assembly and alignment of Sanger sequences was determined using Genious software (version 6.1.5) (Biomatters, Auckland, New Zealand).

### Analysis, classification and comparison of CRISPR, plasmid, and prophage content in *C. autoethanogenum* and other fuel-producing Clostridia

The genome of *C. autoethanogenum* (NC_022592) and genome sequences of *C. acetobutylicum* ATCC824 (NC_003030), DSM1731 (NC_015687) and EA2018 (NC_017295), *C. beijerinckii* NCIMB8052 (NC_009617), *C. saccharobutylicum* (NC_022571), *C. saccharoperbutylacetonicum* (NC_020291), *C. cellulolyticum* H10 (NC_011898), *C. cellulovorans* 743B (NC_014393), *C. thermocellum* ATCC27405 (NC_009012) and DSM1313 (NC_017304), *C. phytofermentans* Isdg (NC_010001), *C. ljungdahlii* DSM13528 (NC_014328) and *C. carboxidivorans* (ACVI01000000; ADEK01000000) were retrieved from NCBI Genbank. The genome sequences of all these organisms were analyzed for CRISPR repeats using the PILER algorithm [75] and CRISPRdb [76]. The sequence of plasmids found in *C. acetobutylicum* ATCC824 (NC_001988), DSM1731 (NC_015686 and NC_015688) and EA2018 (CP002119), *C. saccharoperbutylacetonicum* (NC_020292), and *C. carboxidivorans* (NC_014565) were also analyzed for the presence of CRISPR loci. The repeat sequences detected by PILER and CRISPRdb were combined and manually compared at sequence level. Degenerated CRISPR repeats with several mismatches were not taken into account. The genome sequences of these species were also analyzed for prophage regions using PHAST [86], Phage_Finder [87] and the outputs program manually analyzed. Multiple sequence alignment of repeats and their sequence logos were generated using CLUSTALW and Weblogo. Phylogenetic analyses based on 16S rRNA and *cas1* genes were made using Geneious software. The phylogenetic tree was constructed using the neighbor-joining method with 100 bootstrap steps.

### RT-PCR

RT-PCR was performed to study the expression and operon structure of *cas* genes and the expression CRISPR arrays. Briefly, RNA was isolated (RNeasy Mini Kit, Qiagen, Valencia, CA, USA) from 20 mL *C. autoethanogenum* culture growing in serum bottles at an optical density (OD)_600_ of 0.2 in PETC media and steel mill waste gas (composition: 42% CO, 36% N_2_, 20% CO_2_, and 2% H_2_; collected from a New Zealand Steel site in Glenbrook, New Zealand) as the sole energy and carbon source. cDNA was synthesized using 500 ng of DNaseI (Ambion Inc., Austin, TX, USA)-treated RNA, Superscript III reverse transcriptase and random primers (Life Technologies, Grand Island, NY, USA). PCR was set with 30 ng cDNA- and DNaseI-treated RNA (control) as templates and iproof DNA polymerase (Biorad, Hercules, CA, USA). The primers used in this study are listed (Additional file 9).

### RNA-Seq

RNA-Seq was performed from *C. autoethanogenum* growing in continuous culture in a 1.5-L continuous-stirred tank reactor (CSTR) with steel mill waste gas (composition: 42% CO, 36% N_2_, 20% CO_2_, and 2% H_2_; collected from a New Zealand Steel site in Glenbrook, New Zealand) as the sole energy and carbon source as described previously [52]. A 20-ml sample was centrifuged at 4,000 × rpm for 10 minutes at 4°C. The supernatant was discarded and the pellet was stabilized adding 5 ml of RNAlater® (Ambion Inc). Total RNA was isolated from the cell pellet using RiboPureTM-Bacteria Kit (Ambion Inc.) according to the manufacturer’s standard protocol. DNA was removed using the TURBO DNA-free kit (Ambion Inc.) and RNA quality was assessed using a 2100 bioanalyzer (Agilent Technologies). RNA concentration was determined with a nanodrop 2000 (Thermo Fischer Scientific, Waltham, MA, USA). Ribodepletion was conducted using MICROBEExpressTM kit (Ambion Inc.). cDNA libraries were prepared and sequenced by standard procedures using SOLiD2 sequencing technology. Output from the SOLID run was processed in LifeScope v2.5.1., as specified by the manufacturer (http://downloads.lifetechnologies.com/Analysis_Software/GS/LifeScope/v2.5.1/LifeScope-v2.5.1_4476538_AUG.pdf). Processed reads were mapped to a reference assembly, and the resulting BAM files were imported, displayed, and manually inspected in the Geneious genome browser, v7.0.3 (Biomatters).

## Abbreviations

ABE: acetone-butanol-ethanol; bp: base pairs; CGAL: computing genome assembly likelihoods; CO: carbon monoxide; CODH/ACS: carbon monoxide dehydrogenase/acetyl-CoA synthase; COG: Clusters of Orthologous Groups; CRISPR: clustered regularly interspaced short palindromic repeats; cas: CRISPR-associated; crRNA: CRISPR RNA; FCD: fragment coverage distribution; IS: insertion sequence; NAPDH: nicotinamide adenine dinucleotide phosphate-oxidase; PGM: Personal Genome Machine; RAMP: repeat-associated mysterious proteins; RT-PCR: reverse transcriptase PCR; SMRT: single molecule real-time sequencing; TCA: tricarboxcylic acid cycle.

## Competing interests

LanzaTech NZ, Ltd has a commercial interest in *Clostridium autoethanogenum*.

## Authors’ contributions

SDB, SU, WM, ML, AD and MK carried out bioinformatics analysis. SDB, SN, SU, WM and MK conceived and designed the study and prepared the manuscript. SN carried out the work around the CRISPR system and RT-PCR. SU performed *de novo* and hybrid assemblies and evaluations, OrthoMCL analysis, and ring figure creation, and assisted with PCR. SDT prepared genomic DNA. SS carried out the RNA-Seq experiment. All authors read and approved the final manuscript.

## Authors’ information

Steven D. Brown: corresponding author for DNA sequencing and comparative genomics

Michael Köpke: corresponding author for *Clostridium autoethanogenum*

## Supplementary Material

Additional file 1 **Examples of preliminary PCR and Sanger sequencing studies to close DSM 10061 genome compared to PacBio assembly.** Small regions of overlap in the hybrid assembly weakly joined contigs, and were supported by PCR and Sanger data, but had insufficient support for the Newbler assembly to join contigs (**A**), PCR and Sanger data joined small gaps between contigs in line with predictions using *C. ljungdahlii* DSM 13528 as a reference (**B**), and in other examples much larger products were obtained compared to the predicted PCR product sizes (**C**). Click here for file

Additional file 2 **Computing genome assembly likelihoods (CGAL) results.** CGAL scores for *C. autoethanogenum* DSM 10061 assemblies. Click here for file

Additional file 3 **QUAST results.** QUAST analysis of *C. autoethanogenum* DSM 10061 assemblies. Click here for file

Additional file 4 **REAPR results.** REAPR analysis of *C. autoethanogenum* DSM 10061 assemblies. Click here for file

Additional file 5 **Genome statistics.** General genome statistics for DSM 10061 PacBio assembly. Click here for file

Additional file 6 **Clusters of Orthologous Groups (COG) analysis.** Number of genes associated with COG functional categories for DSM 10061 PacBio assembly. Click here for file

Additional file 7 **Identification of *C. autoethanogenum *from *C. ljungdahlii *orthologs.** The OrthoMCL algorithm [49] was used for ortholog analysis. The 1_taxa tab contains putative paralogs, 2_taxa file contains putative orthologs, Unique tabs contains Unique proteins using default setting, and Table 1 file is general descriptor for all proteins. The 427*C. autoethanogenum* from the unique tab were compared to the *C. ljungdahlii* proteome using BLASTP and 221 proteins were considered dissimilar or unique to DSM 10061 based on e-value scores of ≤1e-5. Click here for file

Additional file 8 **RNA-Seq data for Hydrogenase operon CAETHG_1575-78 and clustered regularly interspaced short paloindromic repeats-associated (CRISPR-cas) array of *C. autoethanogenum*.** Mapped RNA-Seq reads for (FeFe) hydrogenase operon CAETHG_1575-78 and CRISPR-*cas* system of *C. autoethanogenum*. Processing of crRNA in *C. autoethanogenum*. Click here for file

Additional file 9 **Reverse transcriptase (RT)-PCR data for *C. autoethanogenum *clustered regularly interspaced short paloindromic repeats-associated (CRISPR-*cas*) system.** Organization and expression of the *C. autoethanogenum* CRISPR system as determined by RT-PCR and primer list for RT-PCR. Click here for file

Additional file 10 **clustered regularly interspaced short paloindromic repeats (CRISPR) arrays and prophage regions in analyzed genomes.** Overview of position and sequences of identified CRISPR arrays and prophages in industrial relevant Clostridia. Click here for file

Additional file 11 **Graphical representation of clustered regularly interspaced short paloindromic repeats-associated (CRISPR-*cas*) loci in *Clostridium *** **species.** Click here for file

Additional file 12 **Phylogenetic classification of industrial relevant Clostridia based on 16S rRNA and clustered regularly interspaced short paloindromic repeats (CRISPR) systems.** Classification of the CRISPR-*cas* system of *C. autoethanogenum* and phylogenetic classification based on 16S rRNA and *cas1* genes. Click here for file
