# Supplementary figures and images for: Comparison of single-molecule sequencing and hybrid approaches for finishing the genome of Clostridium autoethanogenum and analysis of CRISPR systems in industrial relevant Clostridia
Source: Biotechnol Biofuels. 2014 Mar 21;7:40. doi: 10.1186/1754-6834-7-40 (PMC4022347; doi:10.1186/1754-6834-7-40)

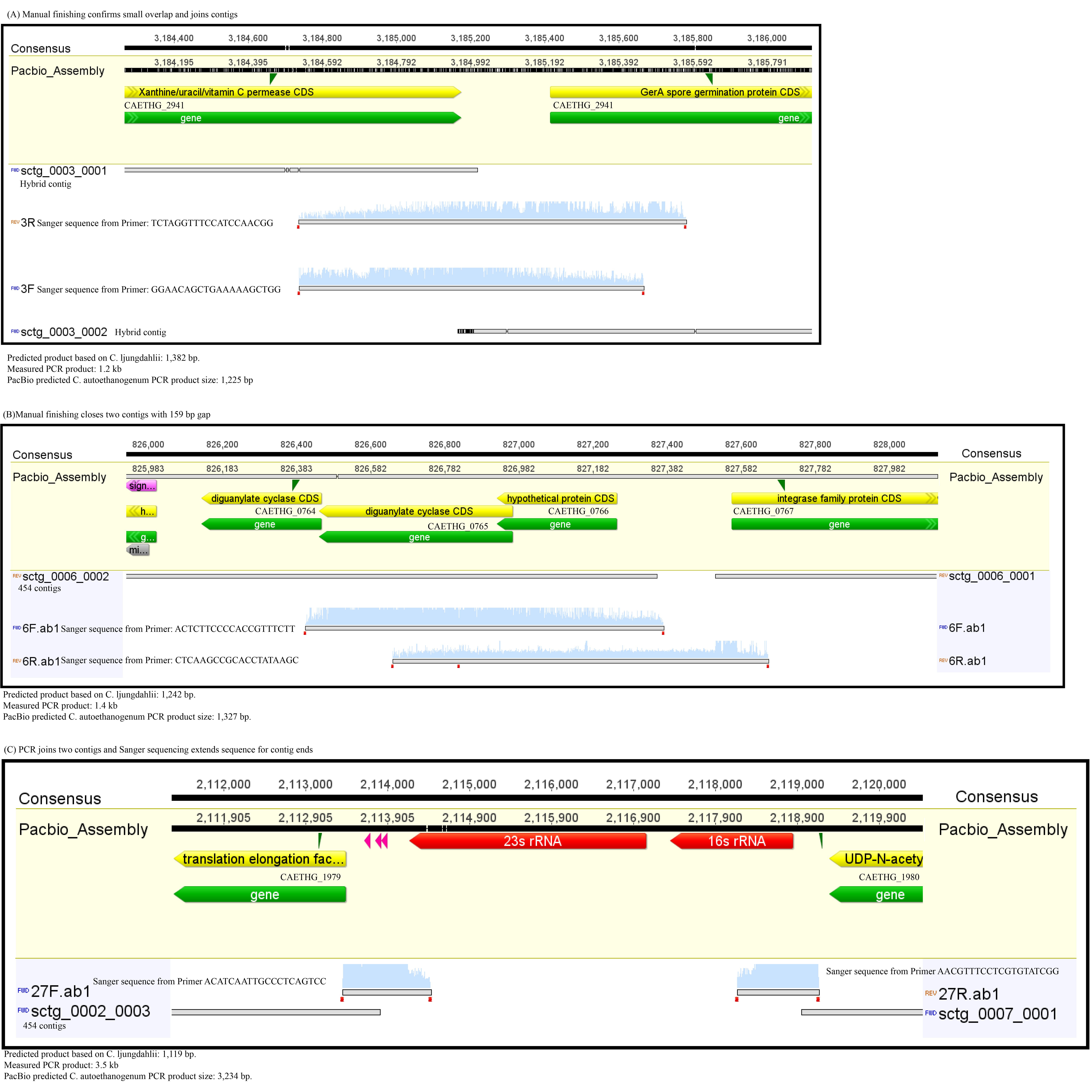

Supplement: Additional file 1 — Examples of preliminary PCR and Sanger sequencing studies to close DSM 10061 genome compared to PacBio assembly. Small regions of overlap in the hybrid assembly weakly joined contigs, and were supported by PCR and Sanger data, but had insufficient support for the Newbler assembly to join contigs (A), PCR and Sanger data joined small gaps between contigs in line with predictions using C. ljungdahlii DSM 13528 as a reference (B), and in other examples much larger products were obtained compared to the predicted PCR product sizes (C). [file 1754-6834-7-40-S1.png]
