# Supplementary material for: Comparison of single-molecule sequencing and hybrid approaches for finishing the genome of Clostridium autoethanogenum and analysis of CRISPR systems in industrial relevant Clostridia
Source: Biotechnol Biofuels. 2014 Mar 21;7:40. doi: 10.1186/1754-6834-7-40 (PMC4022347; doi:10.1186/1754-6834-7-40)
Supplement: Additional file 2 — Computing genome assembly likelihoods (CGAL) results. CGAL scores for C. autoethanogenum DSM 10061 assemblies. [file 1754-6834-7-40-S2.docx]

**Additional file 2. CGAL [**[**1**](#_ENREF_1)**] version 0.9.6 results for *Clostridium autoethanogenum* DSM 10061 assemblies.**

| Assembly | CGAL_Score | CGAL_Score (formatted) |
| --- | --- | --- |
| Illumina_Only | -49339432.8 | -4.93E+07 |
| 454_Hybrid | -52049311.37 | -5.20E+07 |
| 454_Only | -52511662.82 | -5.25E+07 |
| Draft | -54157668.31 | -5.42E+07 |
| Pacbio | -56209788.73 | -5.62E+07 |

1. Rahman A, Pachter L: **CGAL: computing genome assembly likelihoods**. *Genome Biol* 2013, **14**(1):R8.
