# Supplementary material for: Comparison of single-molecule sequencing and hybrid approaches for finishing the genome of Clostridium autoethanogenum and analysis of CRISPR systems in industrial relevant Clostridia
Source: Biotechnol Biofuels. 2014 Mar 21;7:40. doi: 10.1186/1754-6834-7-40 (PMC4022347; doi:10.1186/1754-6834-7-40)
Supplement: Additional file 3 — QUAST results. QUAST analysis of C. autoethanogenum DSM 10061 assemblies. [file 1754-6834-7-40-S3.docx]

**Additional file 3 QUAST [**[**1**](#_ENREF_1)**] version 2.2 results for *Clostridium autoethanogenum* DSM 10061 assemblies.**

| Assembly | Illumina_Only | NCBI_Draft | 454Only | 454Hybrid |
| --- | --- | --- | --- | --- |
| # contigs (>= 0 bp) | 57 | 100 | 32 | 22 |
| # contigs (>= 1000 bp) | 47 | 96 | 30 | 21 |
| Total length (>= 0 bp) | 4311676 | 4323309 | 4305482 | 4308316 |
| Total length (>= 1000 bp) | 4303892 | 4319422 | 4303912 | 4307500 |
| # contigs | 57 | 100 | 32 | 22 |
| Total length | 4311676 | 4323309 | 4305482 | 4308316 |
| Largest contig | 460940 | 436795 | 639527 | 1137625 |
| Reference length | 4352205 | 4352205 | 4352205 | 4352205 |
| GC (%) | 30.92 | 30.97 | 30.91 | 30.92 |
| Reference GC (%) | 31.09 | 31.09 | 31.09 | 31.09 |
| N50 | 255482 | 115901 | 330116 | 687076 |
| NG50 | 255482 | 115901 | 330116 | 687076 |
| N75 | 114708 | 65006 | 110889 | 224907 |
| NG75 | 114708 | 64087 | 110889 | 224907 |
| L50 | 7 | 12 | 5 | 3 |
| LG50 | 7 | 12 | 5 | 3 |
| L75 | 12 | 23 | 11 | 6 |
| LG75 | 12 | 24 | 11 | 6 |
| # misassemblies | 3 | 0 | 0 | 0 |
| Misassembled contigs length | 362096 | 0 | 0 | 0 |
| # local misassemblies | 3 | 0 | 0 | 0 |
| # unaligned contigs | 2 + 0 part | 21 + 1 part | 1 + 0 part | 1 + 0 part |
| Unaligned contigs length | 11033 | 39942 | 5499 | 5499 |
| Genome fraction (%) | 98.764 | 98.407 | 98.784 | 98.853 |
| Duplication ratio | 1.001 | 1 | 1 | 1 |
| # N's per 100 kbp | 0 | 0 | 0.12 | 0 |
| # mismatches per 100 kbp | 1.26 | 0.16 | 1.37 | 0.98 |
| # indels per 100 kbp | 5.65 | 6.07 | 6.65 | 6.16 |
| Largest alignment | 460756 | 436795 | 639307 | 1137445 |
| NA50 | 246708 | 115901 | 330116 | 687032 |
| NGA50 | 246708 | 115901 | 330116 | 687032 |
| NA75 | 112457 | 65006 | 110889 | 224907 |
| NGA75 | 112457 | 64087 | 110889 | 224907 |
| LA50 | 7 | 12 | 5 | 3 |
| LGA50 | 7 | 12 | 5 | 3 |
| LA75 | 13 | 23 | 11 | 6 |
| LGA75 | 13 | 24 | 11 | 6 |

1. Gurevich A, Saveliev V, Vyahhi N, Tesler G: **QUAST: quality assessment tool for genome assemblies**. *Bioinformatics* 2013, **29**(8):1072-1075.
