# Supplementary material for: Comparison of single-molecule sequencing and hybrid approaches for finishing the genome of Clostridium autoethanogenum and analysis of CRISPR systems in industrial relevant Clostridia
Source: Biotechnol Biofuels. 2014 Mar 21;7:40. doi: 10.1186/1754-6834-7-40 (PMC4022347; doi:10.1186/1754-6834-7-40)
Supplement: Additional file 4 — REAPR results. REAPR analysis of C. autoethanogenum DSM 10061 assemblies. [file 1754-6834-7-40-S4.docx]

**Additional file 4. REAPR [**[**1**](#_ENREF_1)**] version 1.0.16 results for *Clostridium autoethanogenum* DSM 10061 assemblies (**Descriptions of errors below).

| **Assembly** | **Total Length** | **Gaps** | **Total Gap Length** | **Original Cotigs** | **Original N50** | **Corrected Contigs** | **Corrected N50** | **Detected Errors** | **FCD Errors** | **Low Coverage Error** | **Error Free Bases (%)** | **Warnings and Notes** |
| --- | --- | --- | --- | --- | --- | --- | --- | --- | --- | --- | --- | --- |
| Illumina_  only | 4311676 | 1 | 512 | 57 | 255482 | 57 | 255482 | 2 | 1 | 1 | 97.23 | 182 warnings:  Low score regions: 0  Links: 95  Soft clip: 2  Collapsed repeats: 4  Low read coverage: 0  Low perfect coverage: 81  Wrong read orientation: 0 |
| NCBI_Draft | 4323309 | 0 | 0 | 100 | 115901 | 100 | 115901 | 0 | 0 | 0 | 97.02 | 190 warnings: Low score regions: 0 Links: 112 Soft clip: 2 Collapsed repeats: 4 Low read coverage: 0 Low perfect coverage: 70 Wrong read orientation: 2 |
| 454_Hybrid | 4308316 | 0 | 0 | 22 | 687076 | 22 | 687076 | 2 | 2 | 0 | 98.6 | 83 warnings: Low score regions: 0 Links: 29 Soft clip: 4 Collapsed repeats: 5 Low read coverage: 0 Low perfect coverage: 45 Wrong read orientation: 0 |
| Pacbio | 4352267 | 0 | 0 | 1 | 4352267 | 1 | 4352267 | 1 | 1 | 0 | 98.44 | 96 warnings: Low score regions: 1 Links: 0 Soft clip: 0 Collapsed repeats: 0 Low read coverage: 1 Low perfect coverage: 94 Wrong read orientation: 0 FCD Error Location for PacBio: 3872494-3873407 - (913 bp) Coverage @ this region Illumina - 40x 454 - 19x  Pacbio 108x Sanger_Coverage - first 392 bp |

See definitions below.


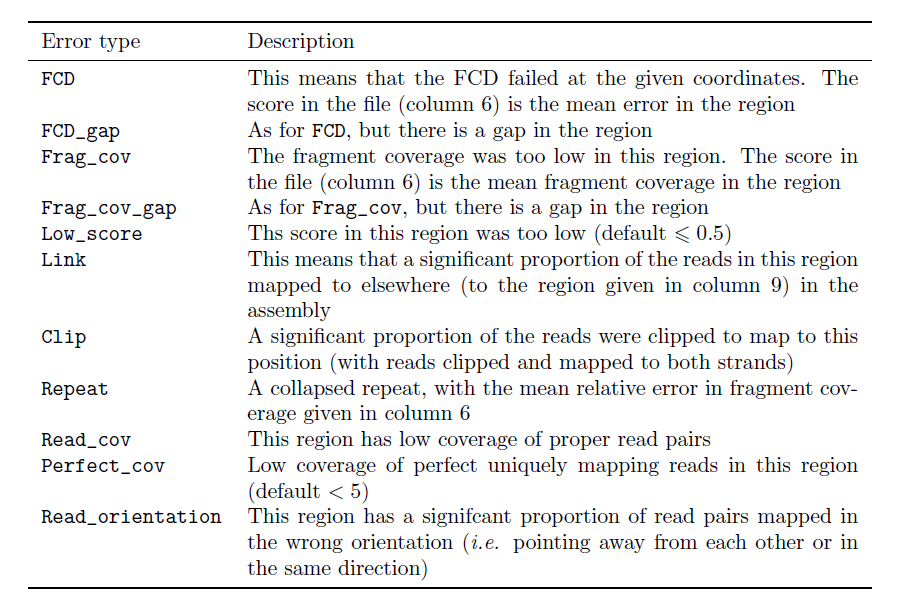


1. Hunt M, Kikuchi T, Sanders M, Newbold C, Berriman M, Otto T: **REAPR: a universal tool for genome assembly evaluation**. *Genome Biology* 2013, **14**(5):R47.
