# Supplementary material for: Comparison of single-molecule sequencing and hybrid approaches for finishing the genome of Clostridium autoethanogenum and analysis of CRISPR systems in industrial relevant Clostridia
Source: Biotechnol Biofuels. 2014 Mar 21;7:40. doi: 10.1186/1754-6834-7-40 (PMC4022347; doi:10.1186/1754-6834-7-40)
Supplement: Additional file 5 — Genome statistics. General genome statistics for DSM 10061 PacBio assembly. [file 1754-6834-7-40-S5.docx]

**Genome statistics**

| **Attribute** | **Value** | **% of Total** |
| --- | --- | --- |
| Genome size (bp) | 4,352,205 | 100% |
| DNA coding region(bp) | 3,679,866 | 84.6% |
| DNA G+C content (bp) | 1,352,824 | 31.1% |
| DNA scaffolds | 1 | 100.0% |
| CRISPR Count | 3 |  |
| Insertion Sequences | 4 |  |
| Riboswitches | 27 |  |
| Cobalamin | 7 |  |
| FMN | 3 |  |
| SAM | 12 |  |
| TPP | 5 |  |
|  |  |  |
| Total genes | 4,161 | 100.0% |
| Protein coding genes | 4,042 | 97.1% |
| Pseudo genes | 18 | 0.4% |
| RNA genes | 101 | 2.4% |
|  |  |  |
| rRNA genes | 27 | 0.6% |
| 5S rRNA | 9 | 0.2% |
| 16S rRNA | 9 | 0.2% |
| 23S rRNA | 9 | 0.2% |
| tRNA genes | 67 | 1.6% |
| Other RNA genes (inc. tmRNA, RNaseP, SRP RNA, and 6S) | 7 | 0.2% |
| Genes with function prediction | 3,283 | 78.9% |
| Genes assigned to COGs | 2,722 | 65.4% |
| Genes with Pfam domains | 3,136 | 75.4% |
| Genes with signal peptides | 242 | 5.8% |
| Genes with transmembrane helices | 1,092 | 26.2% |

The total is based on either the size of the genome in base pairs or the protein coding genes in the annotated genome.
