# Supplementary material for: Comparison of single-molecule sequencing and hybrid approaches for finishing the genome of Clostridium autoethanogenum and analysis of CRISPR systems in industrial relevant Clostridia
Source: Biotechnol Biofuels. 2014 Mar 21;7:40. doi: 10.1186/1754-6834-7-40 (PMC4022347; doi:10.1186/1754-6834-7-40)
Supplement: Additional file 6 — Clusters of Orthologous Groups (COG) analysis. Number of genes associated with COG functional categories for DSM 10061 PacBio assembly. [file 1754-6834-7-40-S6.docx]

| Additional file 6. Number of genes associated with COG functional categories | | | |
| --- | --- | --- | --- |
| Code | Value | % | Description |
| J | 239 | 5.9 | Translation, Ribosomal Structure and Biogenesis |
| K | 451 | 11.2 | Transcription |
| L | 221 | 5.5 | DNA Replication, Recombination and Repair |
| B | 6 | 0.1 | Chromatin structure and dynamics |
| Cellular processes | | | |
| D | 146 | 3.6 | Cell Division and Chromosome Partitioning |
| V | 155 | 3.8 | Defense mechanisms |
| T | 342 | 8.5 | Signal Transduction Mechanisms |
| M | 381 | 9.4 | Cell Envelope Biogenesis, Outer Membrane |
| N | 193 | 4.8 | Cell Motility and Secretion |
| U | 75 | 1.9 | Intracellular trafficking and secretion |
| O | 234 | 5.8 | Posttranslational Modification, Protein Turnover, Chaperones |
| Metabolism | | | |
| C | 458 | 11.3 | Energy production and Conversion |
| G | 341 | 8.4 | Carbohydrate Transport and Metabolism |
| E | 584 | 14.5 | Amino Acid Transport and Metabolism |
| F | 155 | 3.8 | Nucleotide Transport and Metabolism |
| H | 366 | 9.1 | Coenzyme Metabolism |
| I | 93 | 2.3 | Lipid Metabolism |
| P | 311 | 7.7 | Inorganic Ion Transport and Metabolism |
| Q | 226 | 5.6 | Secondary metabolites biosynthesis, transport and catabolism |
| Poorly characterized | | | |
| R | 737 | 18.3 | General Function Prediction Only |
| S | 305 | 7.6 | Function Unknown |
