# Supplementary material for: Comparison of single-molecule sequencing and hybrid approaches for finishing the genome of Clostridium autoethanogenum and analysis of CRISPR systems in industrial relevant Clostridia
Source: Biotechnol Biofuels. 2014 Mar 21;7:40. doi: 10.1186/1754-6834-7-40 (PMC4022347; doi:10.1186/1754-6834-7-40)
Supplement: Additional file 8 — RNA-Seq data for Hydrogenase operon CAETHG_1575-78 and clustered regularly interspaced short paloindromic repeats-associated (CRISPR-cas) array of C. autoethanogenum. Mapped RNA-Seq reads for (FeFe) hydrogenase operon CAETHG_1575-78 and CRISPR-cas system of C. autoethanogenum. Processing of crRNA in C. autoethanogenum. [file 1754-6834-7-40-S8.docx]

**Additional file 8. RNAseq data for [FeFe] hydrogenase operon CAETHG_1575-78 and CRISPR-cas array of *C. autoethanogenum* and processing of crRNA in *C. autoethanogenum*.**

*
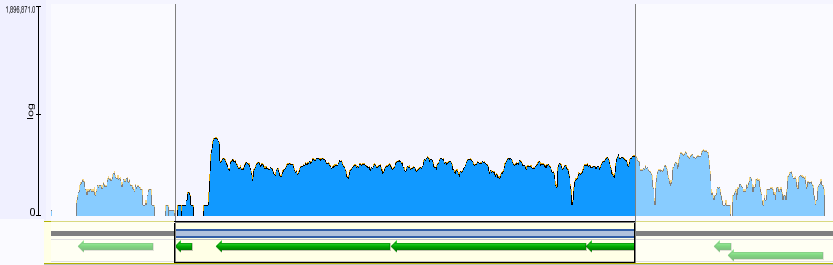
*

CAETHG_1575 CAETHG_1575 CAETHG_1577 CAETHG_1578

Mapped RNAseq reads for [FeFe] hydrogenase operon CAETHG_1575-78 (highlighted) and surrounding genes of *C. autoethanogenum*


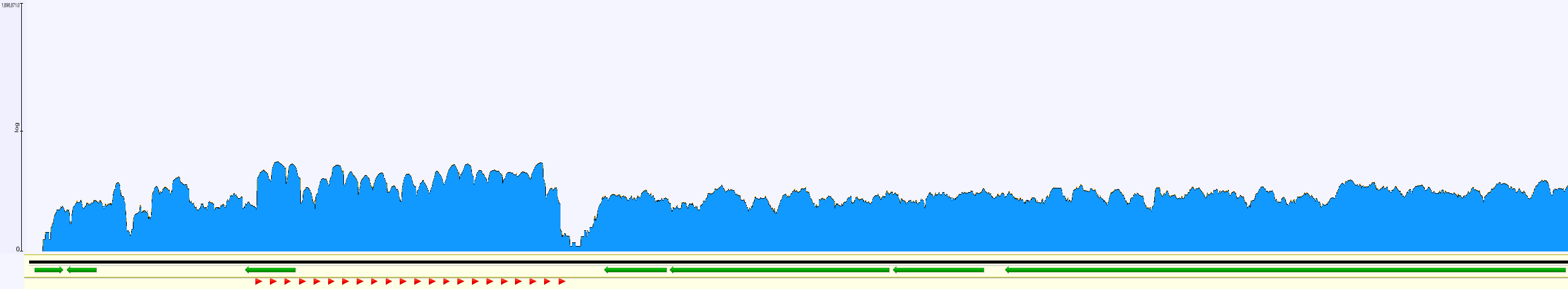


CRISPR Array 1  *cas2 cas1 cas4* *cas3*

*
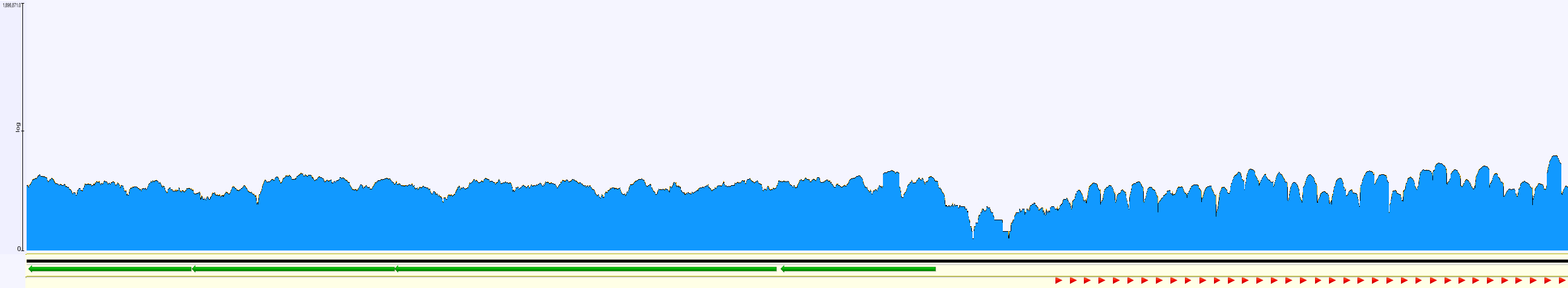
*

*cas5 cas7 cas8b cas6* CRISPR Array 2


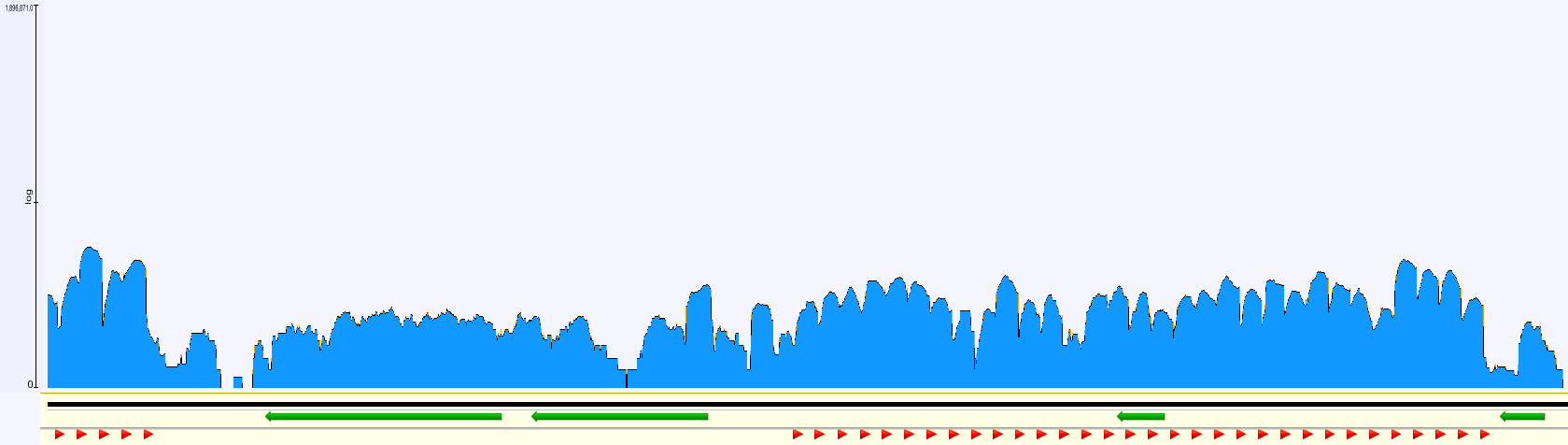


CRISPR Array 2 CRISPR Array 3

Mapped RNAseq reads for CRISPR-cas loci of *C. autoethanogenum*


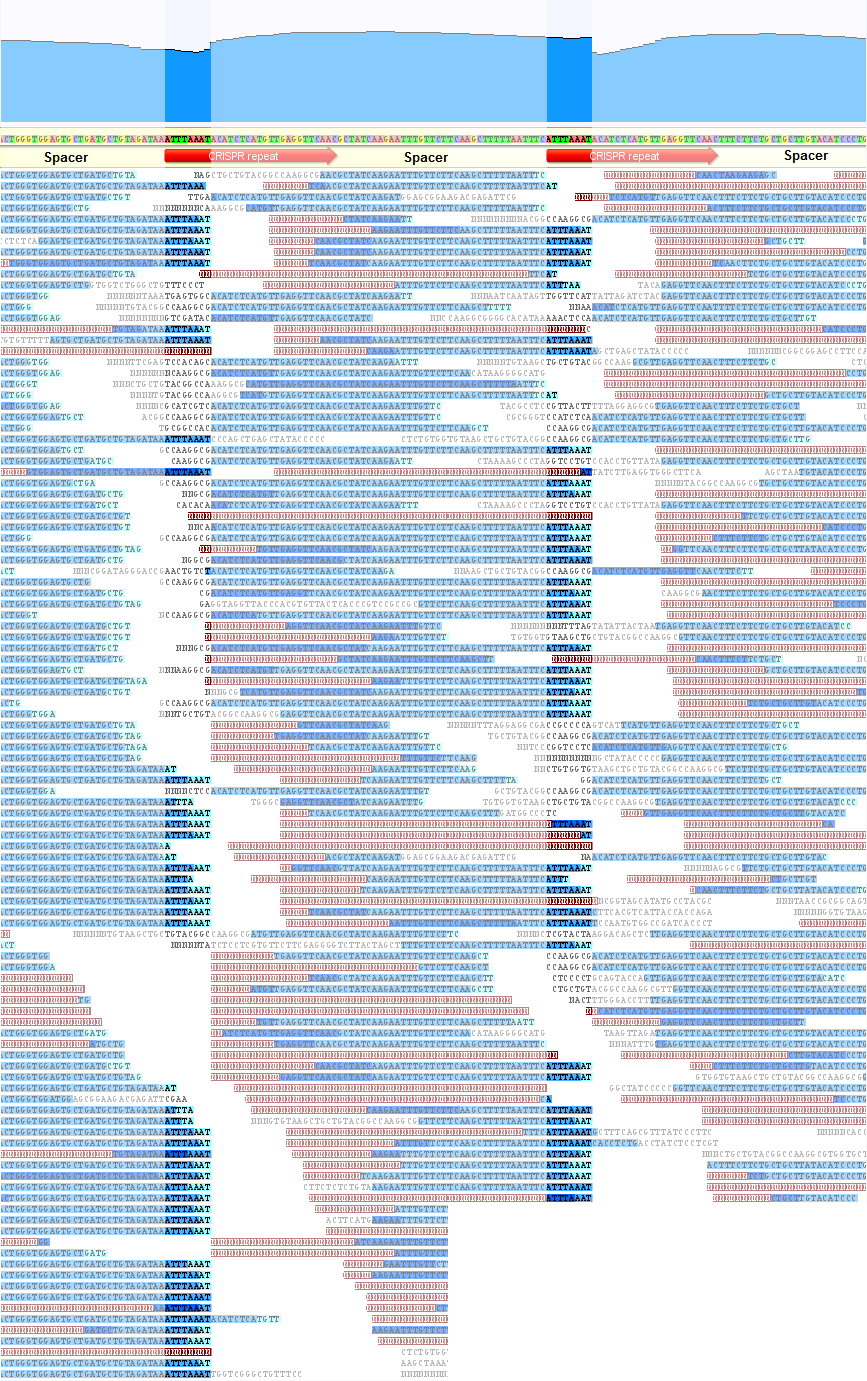


Maped reades of crRNA transcripts of the repeat-spacer region in one of the three CRISPR arrays in *C. autoethanogenum* as an example. The highlighted region indicates the defined 5’-handle in processed crRNAs. Processed crRNAs with 5’ –ATTTAAAT and varying 3’ end.


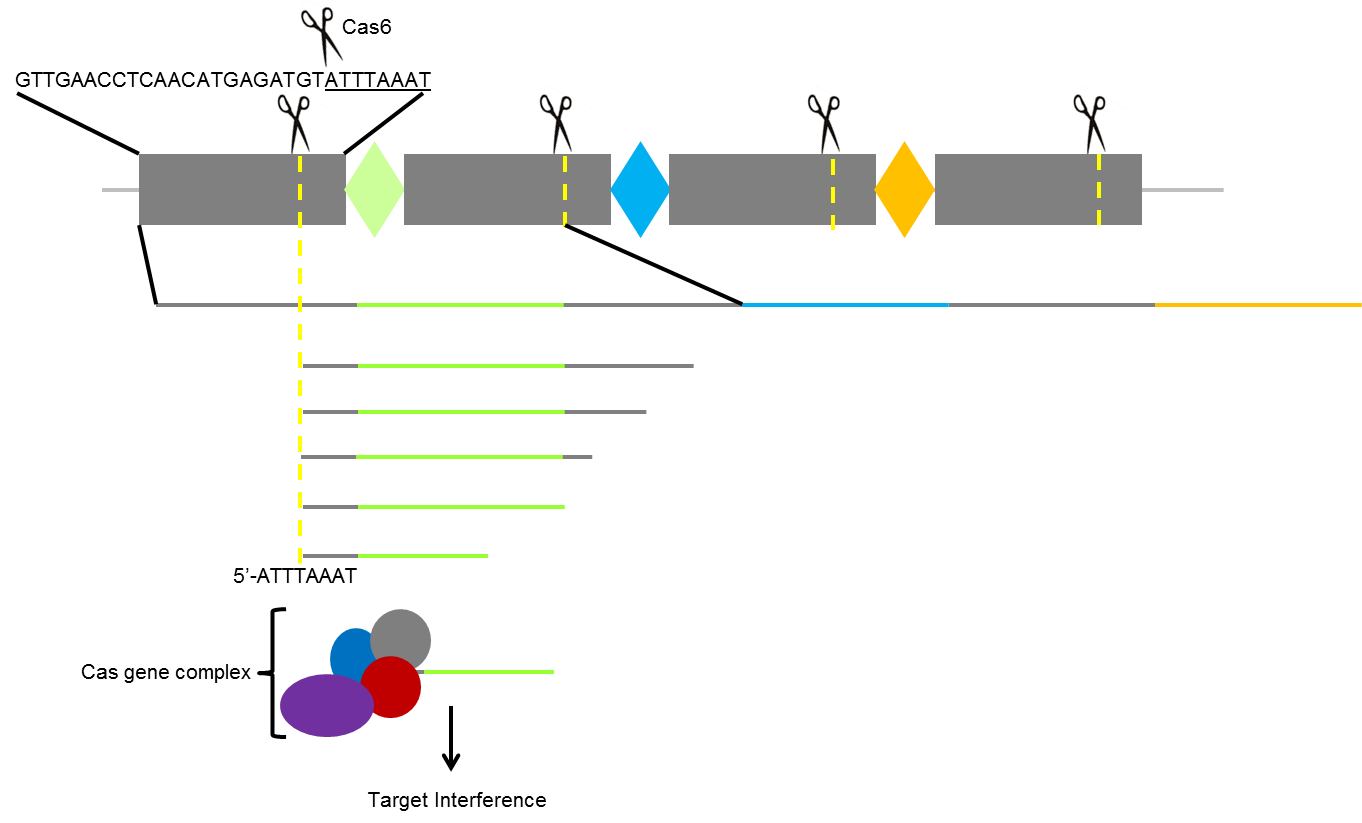


Scheme showing the processing of crRNA in *C. autoethanogenum*. The cleavage site as identified by RNA-Seq with in the repeat (grey rectangles and lines) sequence is indicated by yellow dotted line. Colored rhomboids and lines represent unique spacers. The processing of crRNA with a defined 5’ handle containing 5’- ATTTAAAT-3’ and a spacer is shown as an example. The 5’ handle is proposed to act as a docking site for recruiting other *cas* genes products involved in target interference.
