# Supplementary material for: Comparison of single-molecule sequencing and hybrid approaches for finishing the genome of Clostridium autoethanogenum and analysis of CRISPR systems in industrial relevant Clostridia
Source: Biotechnol Biofuels. 2014 Mar 21;7:40. doi: 10.1186/1754-6834-7-40 (PMC4022347; doi:10.1186/1754-6834-7-40)
Supplement: Additional file 9 — Reverse transcriptase (RT)-PCR data for C. autoethanogenum clustered regularly interspaced short paloindromic repeats-associated (CRISPR-cas) system. Organization and expression of the C. autoethanogenum CRISPR system as determined by RT-PCR and primer list for RT-PCR. [file 1754-6834-7-40-S9.pdf]

Additional file 9. RT-PCR data for *C. autoethanogenum* CRISPR-cas system.

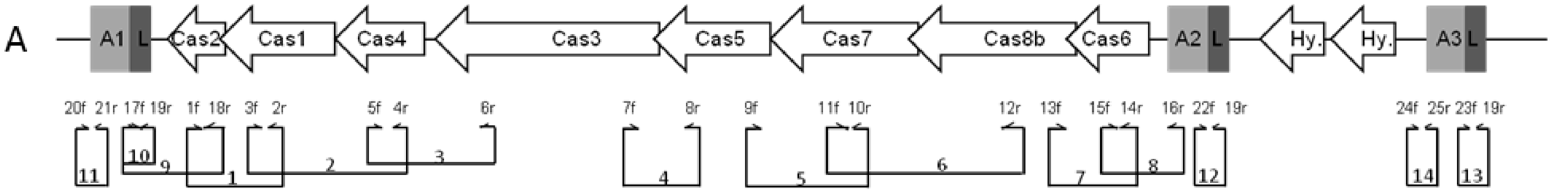

B

```
A1 leader      CACAGTAAAATAGCCATTCCTTATTTTCATTATAACCCTAAAACCCTTAATAATAAAGGC 60
A2 leader      CACAGTAAAATAGCCATTCTTTATTTTCATTATAACCCTAAAACCCTTAACAATAAAGGC 60
A3 leader      CACAGTAAAATAGCCATTTCTTATTTTCATTATAGTCCTAAAACCCTTAATAATAAAGGC 60
               ******************  **************. ************** *********

A1 leader      ATCAACTGAAAATTTTCCAACCGTTTTTCAATTTTCATAAACTCAGCCAAAAAAGTGCCT 120
A2 leader      ATTCGCTGAAAATTTTCCAACCGTTTTTCACTTTTCATAAACTCAGCCAAAAAAATGCCT 120
A3 leader      ATCAACAGAAAATTTTCCAACCGTTTTTCACTTTTCACAAACTCAATAAAAAAAGTACCC 120
               **  ..*:***********************.****** *******. .******.*.**

A1 leader       CAATTCATTACATATCAATGCATTGAAGCACCTTCTATCTTCAAAGAGGTTGGAAAA 177
A2 leader       CAATCCATTACATATCAATGCACTTAAGCACACTTTACCTTCAAAGAGGTTGGAAAA 177
A3 leader       TCATCCATTGAATTCAAATGACTTAAGACATCTCTATCCTCCAAATTAGTTGGAAAA 177
                .** ****..**: .****.. * *..** .    :: ** **** :.*********
```

C

	A1 leader	A2 leader	A3 leader
A1 leader	100	91.1	83.3
A2 leader	91.1	100	82.2
A3 leader	83.3	82.2	100

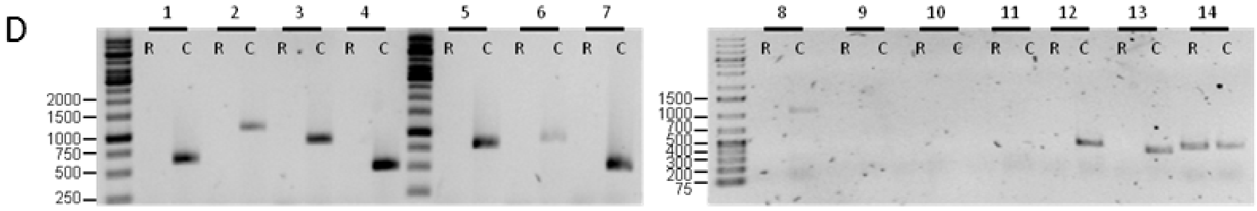

Expression of CRISPR-*cas* loci in *C. autoethanogenum*. (A) The position of primers used in RT-PCR and (B) the agarose gel showing the expression of corresponding PCR products in control (R) and cDNA (C) samples. The numbers in (A) and (B) represent the same primer pairs. (C) Alignment of leader sequence of the three *C. autoethangenum* CRISPR arrays and (D) table showing the per cent sequence identities between them.

List of primers used for RT-PCR in this study.

No.	Primer	Sequence	Amplicon (bp)
1	Cas2F	TTTATCCCTAAAGTATCCTCCTCAAAACAGC	661
2	Cas1R	TCTGCATGAACCATCTGAAGGAAGATT	
3	Cas1F	GCTTGTTTACTTGAATTTGCTTTGTGTTTACC	1250
4	Cas4R	TGAAAGCAGAAGGAAATAAAAATGCAGAA	
5	Cas4F	ACCTGCCATTTTACTGCTTCTACATCTGC	972
6	Cas3R	TGCAGAAGAACAATTTATGGGAAGGG	
7	Cas3F2	TCTCCTGCCGAATTATCCTTGTAGA	600
8	Cas5R2	ATACAGAATACCTTCCTGTGGCTCT	
9	Cas5F	TGCTTATTGATAGTAACTACAGCAACTTTGGC	900
10	Csh2R	GGCAGTGCAGATAGGTCAAGGATTTAATG	
11	Csh2F	GGTTTGCTTCTTCCGCTTCTTTTCC	961
12	Csh1R2	GAGGAAAGAGTATGGATGCAGTTGT	
13	Csh1F2	TACCTTTGTCGGATCTACTGGCTTA	550
14	Cas6R2	TATTGACAGGAGAGGCTGTGGATAA	
15	Cas6F	TTACATCTAAGGGGTACAAGCTGTTGAAGC	1121
16	CRISPR1 distal F	GCAAATACTGATAAAGTAAACAATA	
17	Cas2R	TTCTGTTTTTAGAGGGGCTATAACGACTTCT	738
18	CRIPSR3 proximal R	GGTATAATTTGAAGCTTTTGAAAT	
19	leadR	TGAAAAACGGTTGGAAAATTTTC	475
20	CRIPSR3 proximal R	GGTATAATTTGAAGCTTTTGAAAT	
21	CRISPR3distal R2	CACCTCTTATATAAATAATTCTCTGG	211
22	CRISPR3 distal F2	GCTTGAAGAACAAATTCTTGATA	
19	leadR	TGAAAAACGGTTGGAAAATTTTC	418
23	A2F	TGCACAACTTCGTTAAAACATAATTTAGCG	
19	leadR	TGAAAAACGGTTGGAAAATTTTC	351
24	CRISPR2 distal F	GCGGATCCACAAATATTAAATCA	
25	CRISPR2 proximal F	TCATACAACATTTGTTACGG	392
26	CRISPR2 proximal R	GCAAATACTGATAAAGTAAACAATA	
