# Supplementary material for: Comparison of single-molecule sequencing and hybrid approaches for finishing the genome of Clostridium autoethanogenum and analysis of CRISPR systems in industrial relevant Clostridia
Source: Biotechnol Biofuels. 2014 Mar 21;7:40. doi: 10.1186/1754-6834-7-40 (PMC4022347; doi:10.1186/1754-6834-7-40)
Supplement: Additional file 11 — Graphical representation of clustered regularly interspaced short paloindromic repeats-associated (CRISPR- cas ) loci in Clostridium species. [file 1754-6834-7-40-S11.pdf]

Additional file 11. Graphical representation of CRISPR-*cas* loci in *Clostridium* species. *cas* genes are represented by arrows and CRISPR arrays by shaded rectangles. The classification of *cas*-gene operon where could be identified is indicated. *cas* operons were classified based on a recent classification system [1].

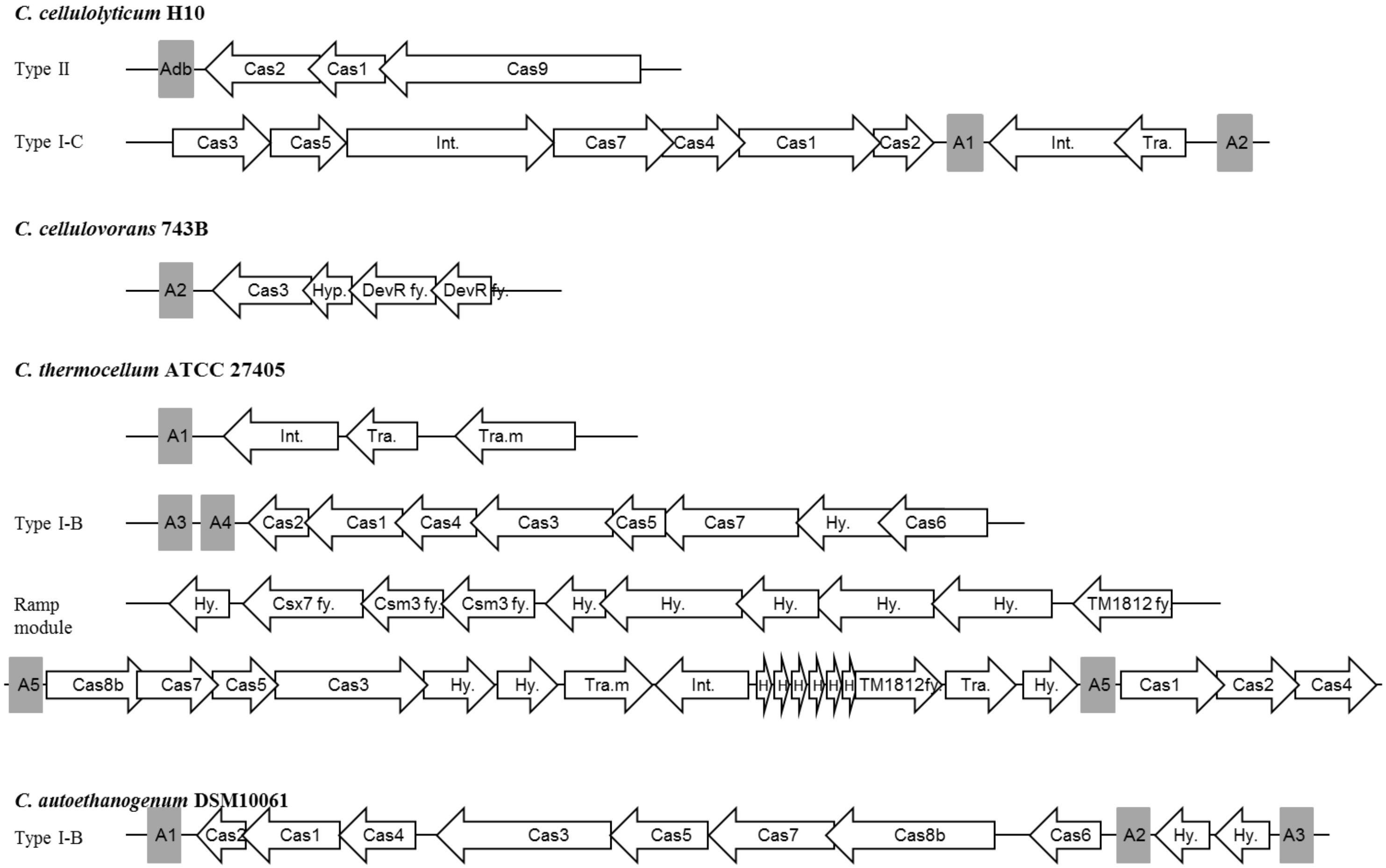

1. Bhaya D, Davison M, Barrangou R: **CRISPR-Cas systems in Bacteria and Archaea: Versatile small RNAs for adaptive defense and regulation**. *Ann Rev Genetics* 2011, **45**:273-297.
