# Supplementary material for: Comparison of single-molecule sequencing and hybrid approaches for finishing the genome of Clostridium autoethanogenum and analysis of CRISPR systems in industrial relevant Clostridia
Source: Biotechnol Biofuels. 2014 Mar 21;7:40. doi: 10.1186/1754-6834-7-40 (PMC4022347; doi:10.1186/1754-6834-7-40)
Supplement: Additional file 12 — Phylogenetic classification of industrial relevant Clostridia based on 16S rRNA and clustered regularly interspaced short paloindromic repeats (CRISPR) systems. Classification of the CRISPR-cas system of C. autoethanogenum and phylogenetic classification based on 16S rRNA and cas1 genes. [file 1754-6834-7-40-S12.pdf]

Additional file 12. Phylogenetic classification of industrial relevant Clostridia based on 16s rRNA and CRISPR systems.

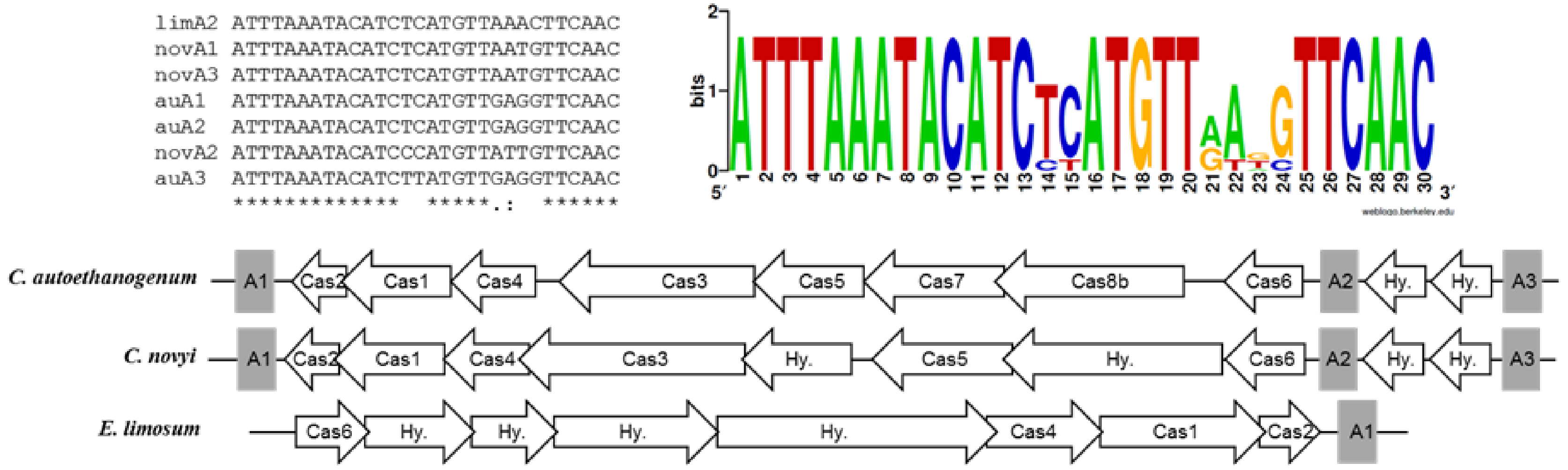

Comparison of CRISPR-*cas* system between *C. autoethanogenum*, *C. novyi* and *E. limosum*. Each section includes alignment of CRISPR repeats, sequence logo and graphic representation of CRISPR-*cas* system.

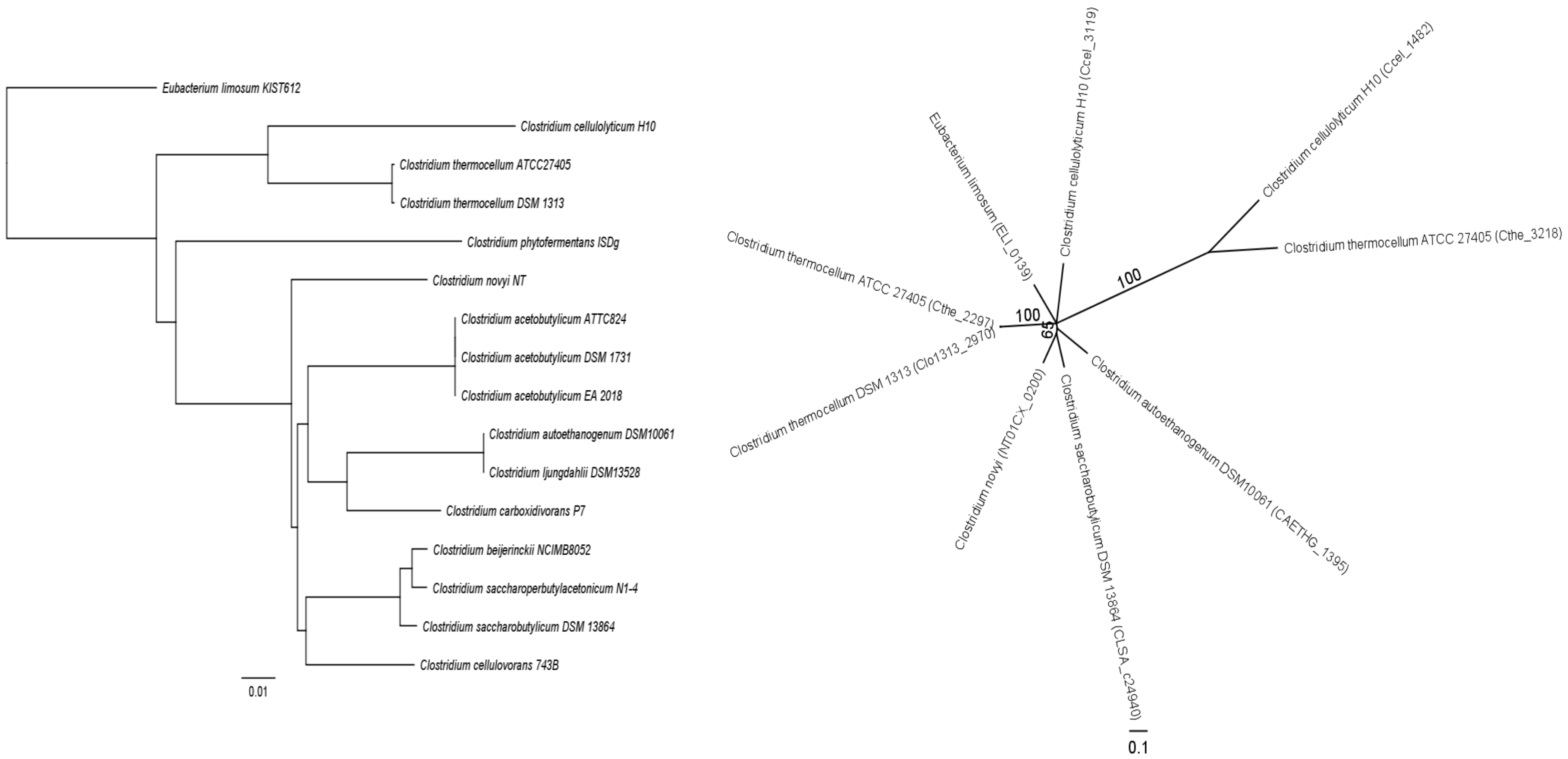

Phylogenetic classification of industrial relevant Clostridia strains based 16S rRNA (left) on *cas1* gene sequences (right). Phylogenetic tree was generated using Geneious software by Neighbor-joining method and the bootstrap values are indicated.
